# Synthesis and Iodine Adsorption Properties of Organometallic Copolymers with Propeller-Shaped Fe(II) Clathrochelates Bridged by Different Diaryl Thioether and Their Oxidized Sulfone Derivatives

**DOI:** 10.3390/polym14224818

**Published:** 2022-11-09

**Authors:** Suchetha Shetty, Noorullah Baig, Bassam Alameddine

**Affiliations:** 1 Department of Mathematics and Natural Sciences, Gulf University for Science and Technology, Hawally 32093, Kuwait; 2 Functional Materials Group—CAMB, GUST, Hawally 32093, Kuwait

**Keywords:** Fe(II) clathrochelate, copolymers, poly(vinylene sulfone), selective oxidation, iodine adsorption, environmental remediation

## Abstract

Three organometallic copolymers, ICP1-3, containing iron(II) clathrochelate units with cyclohexyl lateral groups and interconnected by various thioether derivatives were synthesized. The reaction of the latter into their corresponding OICP1-3 sulfone derivatives was achieved quantitatively using mild oxidation reaction conditions. The target copolymers, ICP1-3 and OICP1-3, were characterized by various instrumental analysis techniques, and their iodine uptake studies disclosed excellent iodine properties, reaching a maximum of 360 wt.% (q_e_ = 3600 mg g^−1^). The adsorption mechanisms of the copolymers were explored using pseudo-first-order and pseudo-second-order kinetic models. Furthermore, regeneration tests confirmed the efficiency of the target copolymers for their iodine adsorption even after several adsorption-desorption cycles.

## 1. Introduction

As the global demand for energy is soaring, alternative sources are being scrutinized to overcome the shortages. Among others, nuclear energy is thought of as a possible source, given its superior energy density and reduced output of greenhouse gases [1]. Nevertheless, a major challenge should be overcome to ensure safe operations in the production of energy from nuclear resources, namely, the proper disposal of radioactive waste [2], whose ^131^I and ^129^I isotopes are the chief gaseous radioactive pollutants originating from uranium fission [3,4]. Despite the relatively short ~8-day half-life (t_1/2_) of ^131^I, it can still be easily absorbed by organisms, accumulating in the thyroid gland and thus resulting in severe internal radiation damage [5,6]. On the other hand, the ultra-long 1.57 × 10^7^ years half-life of ^129^I easily escapes to the biosphere, therefore leading to long-term pollution [7,8,9]. Consequently, the capture of radioiodine isotopes is deemed necessary, and various methods have been developed for this purpose, such as chemical precipitation, dry dedusting and physical adsorption [10,11,12]. The latter technique, which employs porous materials, is considered the most promising due to its high adsorption capacity, versatile use, and economic feasibility [13,14,15,16,17]. As a result, myriad porous materials have been synthesized and tested for iodine capture, including, among others, activated carbon [18], metalorganic framework structures (MOF) [19,20], organic polymers [21,22], covalent organic frameworks (COFs) [23,24] and amorphous materials [25,26].

Iron(II) clathrochelates are robust metalorganic complexes whose intricate structural features and modular synthesis have qualified them to be utilized as potential building blocks in several applications, including as biosensors [27,28], catalysts for hydrogen generation [29,30], materials for electronic transport [31], organogels [32], supramolecular structures [33] and porous materials for the adsorption of different gases and dyes [32,34,35,36,37]. Recently, our group has disclosed various metalorganic polymers containing Fe(II) clathrochelate derivatives, which revealed the efficient uptake of organic dyes and lithium ions from water solutions [38], as well as iodine capture [39]. We show in this work the synthesis of new Fe(II) clathrochelate-based copolymers bearing cyclohexyl side groups and interconnected by thioether aryl derivatives, which reveal very good iodine capture properties of 360 wt.% (q_e_ = 3600 mg g^−1^).

## 2. Materials and Methods

Syntheses of the target compounds were performed under dry argon. 4-Tert-butylphenylacetylene, 4-mercaptophenylboronic acid, 1,2-cyclohexanedione dioxime, and iron(II) chloride were purchased from Merck (Darmstadt, Germany). TC1-3 was prepared by following the procedures mentioned in the literature [38], while all the other chemicals were utilized as received from the suppliers. Anhydrous solvents, in particular, THF, dichloromethane (DCM), chloroform, acetone, diethyl ether, methanol, and hexane, were dehydrated over molecular sieves and deoxygenated by using a positive stream of argon for half an hour. Thin-layer chromatography (TLC) was carried out on Al films coated with silica gel 60F254 and revealed utilizing an ultraviolet lamp. Nuclear magnetic resonance (NMR; ^1^H: 600 MHz, ^13^C:150 MHz) spectra were recorded on a Bruker BioSpin GmbH 600 MHz spectrometer using CD_2_Cl_2_ and DMSO-d_6_ as solvents with the chemical shifts (δ) given in ppm and using tetramethylsilane (TMS) as reference. Nuclear magnetic resonance magic angle spinning solid-state (^13^C: 150 MHz) spectra were recorded on Bruker BioSpin GmbH 600 MHz. Fourier transform infrared spectra were recorded on an Agilent Cary 630 FTIR. Thermogravimetric analysis (TGA) was carried out on a Shimadzu TGA-60H (Kyoto, Japan) analyzer to evaluate the thermal stability of polymers from room temperature to 800 °C using a heating rate of 10 °C/min under an inert atmosphere of nitrogen. X-ray photoelectron spectroscopy (XPS) was carried out utilizing a Thermo ESCALAB 250 Xi with a monochromatic Al Kα-radiation source (1486.6 eV) and a spot aperture of 850 μm. The XPS spectra were acquired and processed using Thermo Advantage software. The XPS chamber base pressure was in the range of 10^−10^ to 10^−9^ torr. The analyzer was operated with a pass energy of 20 eV, a dwell time of 50 min and a step size of 0.1 eV. Electron impact high-resolution mass spectroscopy (EI-HRMS) was recorded using a Thermo DFS and standard perfluorokerosene (PFK) as lock mass. The analyzed data were converted to accurate mass by utilizing X-Calibur accurate mass calculation software.

### 2.1. Synthesis

#### 2.1.1. Synthesis of MTC

4-(4-(Tert-butyl)styryl)thio)phenyl)boronic acid (TC) (0.187 g, 0.60 mmol, 1 eq.), 1,2-cyclohexanedione dioxime (0.111 g, 0.78 mmol, 1.3 eq.) and iron(II) chloride (0.034 g, 0.27 mmol, 0.45 eq.) in deoxygenated methanol (5 mL) were charged in a Schlenk tube under a positive stream of argon. The reaction mixture was refluxed overnight, and the resulting solution was evaporated under reduced pressure. The desired product was isolated by precipitation using DCM-hexane followed by filtration affording a red solid (0.24 g, 86%); ^1^H-NMR (600 MHz, CD_2_Cl_2_, ppm): δ 7.67–7.66 (*d*, 4H, *J = 6 Hz*, ArH), 7.48–7.47 (*d*, 4H, *J = 12 Hz*, ArH), 7.44–7.42 (*brm*, 8H, ArH), 6.57–6.56 (*d*, *J = 6 Hz*, 2H, Vinylic-CH), 6.52–6.50 (*d*, *J = 12 Hz*, 2H, vinylic-CH), 2.92 (*s*, 12H, cyclohexyl-CH_2_), 1.80 (*s*, 12H, cyclohexyl-CH_2_) 1.35 (*brs*, 18H, t-butyl-CH_3_); ^13^C-NMR (150 MHz, CD_2_Cl_2_, ppm): δ 152.62, 150.78, 135.89, 134.50, 133.18, 131.54, 129.55, 129.36, 129.04, 126.99, 126.31, 125.80, 123.63, 35.10, 31.61, 26.82, 22.19; EI-HRMS: m/z calculated for (M^•+^) C_54_H_62_B_2_FeN_6_O_6_S_2_ 1032.3708 found 1032.2835.

#### 2.1.2. Synthesis of ICP1 (Procedure A)

TC1 (0.3 g, 0.49 mmol, 1 eq.), 1,2-cyclohexanedione dioxime (0.21 g, 1.47 mmol, 3 eq.), and iron(II) chloride (0.06 g, 0.49 mmol, 1 eq.) in chloroform (10 mL) were charged in a Schlenk tube, and the reaction mixture was refluxed for 48 h under argon. The resulting solution was evaporated under reduced pressure. The desired product was isolated by precipitation using DCM-hexane followed by filtration, and the precipitate was washed successively with hexane (20 mL), acetone (20 mL), and methanol (20 mL), affording a red solid (0.445 g, 89%). Solid state ^13^C-NMR (150 MHz, ppm): 151.81, 145.24, 133.06, 124.91, 116.10, 51.06, 26.24 and 22.03; FTIR (KBr) (cm^−1^): 3056 (aromatic -C-H stretch), 2946 (aliphatic -C-H stretch.), 1689 (C=N stretch), 1583 (aromatic C=C stretch), 1441 (aliphatic -C-H bend), 961 (alkene C=C bend) and 807 (aromatic C=C bend).

#### 2.1.3. Synthesis of ICP2

ICP2 was prepared following procedure A with: TC2 (0.3 g, 0.49 mmol, 1 eq.), 1,2-cyclohexanedione dioxime (0.211 g, 1.48 mmol, 3 eq.), iron(II) chloride (0.06 g, 0.49 mmol, 1 eq.), and chloroform (10 mL). Red solid (0.45 g, 88%); solid state ^13^C-NMR (150 MHz, ppm): 151.97, 132.82, 129.65, 114.22, 85.74, 66.82, 26.21, 21.79 and 17.63; FTIR (KBr) (cm^−1^): 3042 (aromatic -C-H stretch), 2945 (aliphatic -C-H stretch), 1605 (aromatic C=C stretch), 1442 (aliphatic -C-H bend), 967 (alkene C=C bend), 826 (aromatic C=C bend) and 730 (alkene C=C bend).

#### 2.1.4. Synthesis of ICP3

ICP3 was prepared following procedure A with: TC3 (0.3 g, 0.55 mmol, 1 eq.), 1,2-cyclohexanedione dioxime (0.232 g, 1.63 mmol, 3 eq.), iron(II) chloride (0.069 g, 0.55 mmol, 1 eq.), and chloroform (11 mL). Red solid (0.45 g, 88%). Solid state ^13^C-NMR (150 MHz, ppm): 152.20, 136.92, 133.32, 128.06, 112.49, 46.90, 39.78, 26.50, and 22.07; FTIR (KBr) (cm^−1^): 3064 (aromatic -C-H stretch), 2946 (aliphatic -C-H stretch), 1680 (C=N stretch), 1582 (aromatic C=C stretch), 1441 (aliphatic -C-H bend), 963 (alkene C=C bend) and 812 (aromatic C=C bend).

#### 2.1.5. Synthesis of OMTC (Procedure B)

To a stirring suspension of MTC (50 mg, 0.048 mmol) in acetic acid (5 mL) was added dropwise 4 mL of aqueous hydrogen peroxide (aq. H_2_O_2_, 30 wt%), and the reaction mixture was stirred at 50 °C for 1 h. The resulting red precipitate was filtered off and washed with deionized water (20 mL) and diethyl ether (20 mL), then dried under a vacuum. Red solid (51 mg, 95%). ^1^H-NMR (600 MHz, CD_2_Cl_2_, ppm): δ 7.83–7.80 (*brm*, 4H, ArH), 7.65–7.56 (*brm*, 8H, ArH), 7.49 (*brm*, 4H, ArH), 7.09–7.00 (*brm*, 2H, vinylic-CH), 6.46–6.40 (*brm*, 2H, vinylic-CH), 2.91 (*s*, 12H, cyclohexyl-CH_2_), 1.81 (*s*, 12H, cyclohexyl-CH_2_) 1.36 (*brs*, 18H, t-butyl-CH_3_); ^13^C-NMR (150 MHz, CD_2_Cl_2_, ppm): δ 152.93, 152.35, 152.22, 141.59, 140.52, 137.90, 132.63, 130.42, 129.76, 128.97, 128.31, 125.59, 123.18, 34.71, 30.92, 26.21, 21.56; EI-HRMS: m/z calculated for (M^•+^) C_54_H_62_B_2_FeN_6_O_10_S_2_ 1096.3504 found 1096.2200.

#### 2.1.6. Synthesis of OICP1

OICP1 was prepared following procedure B with ICP1 (0.1 g, 0.098 mmol), acetic acid (7 mL) and 30 wt% aq. H_2_O_2_ (10 mL). Red solid (0.98 g, 92%). FTIR (KBr) (cm^−1^): 3056 (aromatic -C-H stretch), 2946 (aliphatic -C-H stretch), 1588 (aromatic C=C stretch), 1436 (aliphatic -C-H bend), 1313 (O=S=O stretch), 1143 (O=S=O stretch), 964 (alkene C=C bend) and 822 (aromatic C=C bend).

#### 2.1.7. Synthesis of OICP2

OICP2 was prepared following procedure B with: ICP2 (0.15 g, 0.146 mmol), acetic acid (10 mL) and 30 wt% aq. H_2_O_2_ (15 mL). Red solid (0.153 g, 96%). FTIR (KBr) (cm^−1^): 3042 (aromatic -C-H stretch), 2945 (aliphatic -C-H stretch), 1605 (aromatic C=C stretch), 1442 (aliphatic -C-H bend), 1310 (O=S=O stretch), 1120 (O=S=O stretch), 967 (alkene C=C bend), 826 (aromatic C=C bend) and 730 (alkene C=C bend).

#### 2.1.8. Synthesis of OICP3

OICP3 was prepared following procedure B with: ICP3 (0.15 g, 0.157 mmol), acetic acid (10 mL) and 30 wt% aq. H_2_O_2_ (15 mL). Red solid (0.156 g, 98%). FTIR (KBr) (cm^−1^): 3051 (aromatic -C-H stretch), 2954 (aliphatic -C-H stretch), 1601 (aromatic C=C stretch), 1431 (aliphatic -C-H bend), 1311 (O=S=O stretch), 1142 (O=S=O stretch), 964 (alkene C=C bend) and 831 (aromatic C=C bend).

## 3. Results

**Scheme 1:**
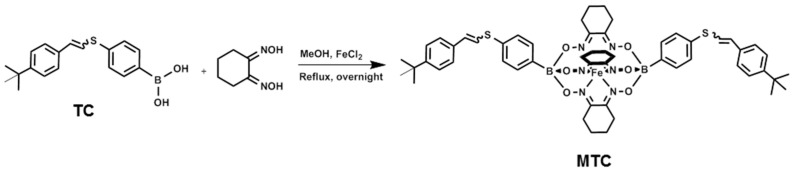
Synthesis of monomer MTC.

The prototypical monomer MTC was synthesized by reacting iron(II) chloride with three equivalents of 1,2-cyclohexanedione dioxime ligand and two equivalents of the capping reagent TC in refluxing methanol overnight under argon, thus affording the desired product in 86% yield (Scheme 1) in high purity as confirmed by ^1^H, ^13^ C NMR, electron impact-HRMS, and Fourier transform IR spectroscopy (Figures S2, S5, S10 and S12 in the Supporting Information File).

**Scheme 2:**
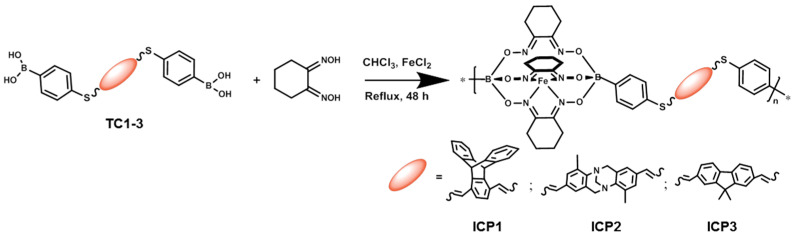
Synthesis of copolymers ICP1-3.

The successful formation of MTC prompted us to make the copolymer derivatives (Scheme 2) using an equimolar amount of iron(II) chloride with one of the three synthons TC1-3 along with three equivalents of 1,2-cyclohexanedione dioxime in boiling chloroform under an inert atmosphere for two days, which yielded the target cyclohexyl-cladded Fe(II) clathrochelate copolymers interconnected by various thioether aryl units ICP1-3 in excellent yields (~89%). The aforementioned copolymers were insoluble; hence, solid-state ^13^C-NMR, XPS spectroscopy, and FTIR were employed to determine their structures in addition to using TGA to assess their thermal stability (Figures S1–S4, S7, S8, S13, S14, S17 and S18 in the Supporting Information File).

**Scheme 3:**
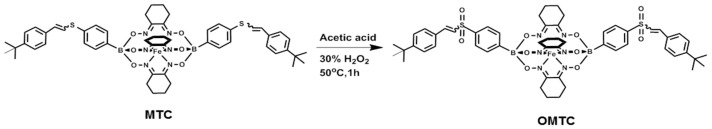
Synthesis of monomer OMTC.

Oxidation of thioether groups in MTC into their corresponding sulfones [40] was carried out using H_2_O_2_ in AcOH at 50 °C for 1 h (Scheme 3), affording the target OMTC quantitatively, which was isolated by filtration and whose structure was confirmed by ^1^H- and ^13^C-NMR, FTIR and XPS spectroscopy (Figures S3, S6, S11 and S12 in the Supporting Information File).

**Scheme 4:**
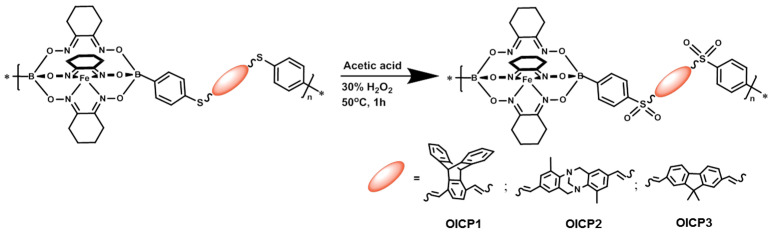
Synthesis of copolymers OICP1-3.

Likewise, Scheme 4 displays the oxidative reaction of the thioether units in copolymers ICP1-3 into their sulfone groups, employing the same abovementioned procedure to synthesize OMTC and thus yielding copolymers OICP1-3 in excellent yields (92–98%). The target copolymers are highly insoluble in common organic solvents; hence, their structures were confirmed by XPS, Fourier transform IR, and TGA.

**Figure 1:**
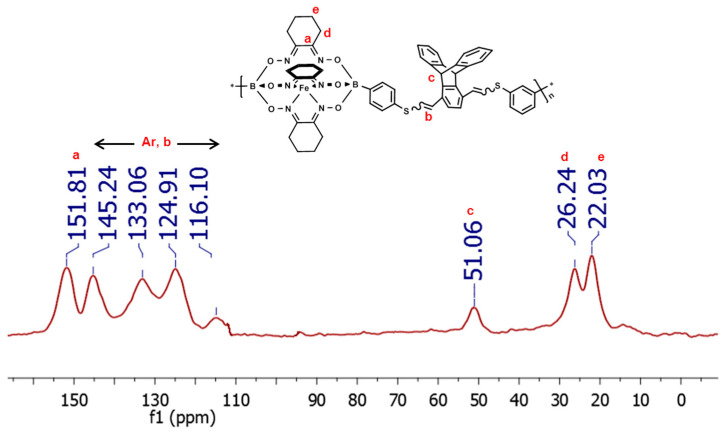
Solid-state ^13^C-NMR spectrum of ICP1.

Figure 1 portrays the SS ^13^C-NMR spectrum of ICP1, where the chemical shift at 151.81 ppm (c.f. Figure 1, peak a) is attributed to C=N carbon atoms of the clathrochelate unit, whereas the chemical shifts at 145.24 ppm, 133.06 ppm, 124.91 ppm and 116.10 ppm correspond to the aromatic and vinylic carbons (c.f. Figure 1, peaks Ar and b). In addition, the peak at 51.06 ppm is assigned to the sp^3^ hybridized carbon atoms of the triptycene unit (c.f. Figure 1, peak c). The peaks observed at 26.24 ppm and 22.03 ppm are related to the methylene carbons of the cyclohexyl groups (c.f. Figure 1, peaks d and e). It should be noted that the solid-state ^13^C-NMR spectra of ICP2,3 reveal the characteristic peaks, which ascertain their structures as well (Figures S7 and S8 in the Supporting Information File).

**Figure 2:**
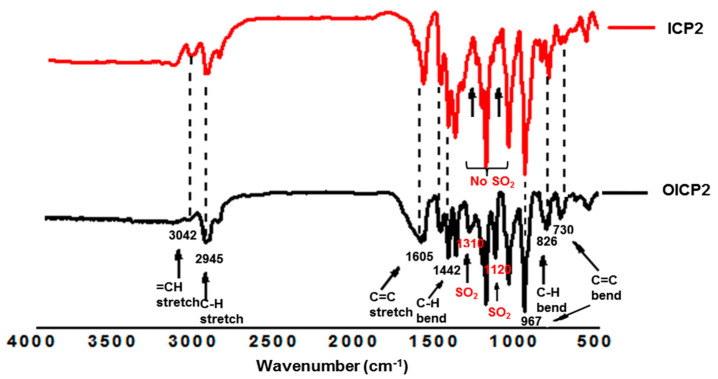
Comparative FTIR spectrum of ICP2 (up) and OICP2 (down).

Figure 2 discloses the comparative Fourier transform IR absorption spectra of the copolymer ICP2 and its respective sulfone derivative OICP2: the non-symmetric and symmetric stretching vibrations of sulfone (O=S=O) in OICP2 were observed at 1310 cm^−^^1^ and 1120 cm^−^^1^, respectively, which strongly denotes the oxidation of sulfur moieties in ICP2 into sulfone in OICP2 [41]. In addition, the aromatic C-H stretching vibration peaks of ICP2 and OICP2 are observed at 3042 cm^−^^1^, whilst the aliphatic C-H stretching vibration peaks were detected at 2945 cm^−^^1^ [42]. The absorption band seen at 1605 cm^−^^1^ is assigned to the aromatic C=C stretching vibrations, while those observed at 1442 cm^−^^1^ and 826 cm^−^^1^ are attributed to the aliphatic and aromatic C-H group bending vibrations, respectively. In addition, the C=C bending vibrations of the conjugated alkene groups are observed at 967 cm^−^^1^ and 730 cm^−^^1^ [43,44,45]. Similarly, all the other target copolymers, ICP1,3 and OICP1,3, reveal their characteristic peaks, therefore proving their successful synthesis (Figures S12–S16 in the Supporting Information File).

**Figure 3:**
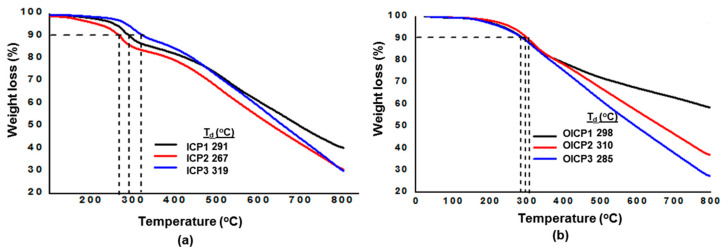
TGA thermograms of copolymers ICP1-3 (**a**) and OICP1-3 (**b**). T_d_ represents the temperature of 10% weight loss.

Thermogravimetric analysis (TGA) of ICP1-3 reveals a 10% decrease in weight at temperatures ranging from 267 °C to 319 °C. Interestingly, oxidation of these latter into their respective copolymers OICP1-3, which bear stiffer sulfone units, leads only to slight variations in the 10% weight loss temperature, whose ranges were detected between 298 °C to 310 °C (Figure 3).

**Figure 4:**
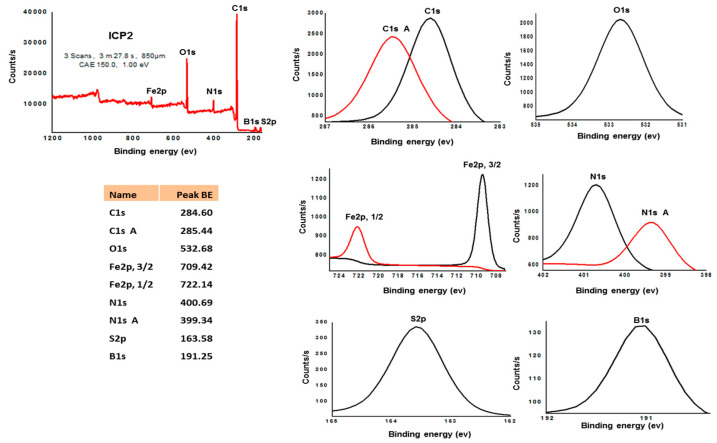
Survey scan high-resolution XPS spectra of C1s, O1s, Fe2p, N1s, S2p, and B1s of ICP2.

X-ray photoelectron spectroscopy (XPS) analysis of copolymers ICP1-3 and OICP1-3 allowed for the determination of their elemental composition as confirmed by the survey scan spectra, which revealed all the elemental peaks (Figures S17–S21 in the Supporting Information File). XPS spectrum of ICP2, depicted in Figure 4, divulges the presence of all the constituting elements, namely, C, O, N, B, S, and Fe [46]. The C1s peak of ICP2 can be integrated into two main binding energies at ~284.60 eV and 285.44 eV, with the former assigned to the conjugated carbon atoms (C=C) while the latter is correlated to that of the imine carbons (C=N). The binding energy observed at ~532.68 eV is attributed to oxygen coupled with both nitrogen and boron. Furthermore, the N1s spectrum exhibits two peaks at 399.34 eV and 400.69 eV, which can be attributed to carbon and nitrogen (C-N) bonds encountered in the Tröger’s base and metalorganic units, respectively. S2p was seen at 163.58 eV, thus indicating the presence of a C-S bond [14,35]. Similarly, the B1s peak was found at 191.25 eV, which clearly confirms the presence of boron oxide (B-O) [47]. Figure 4 also portrays the XPS peak for Fe2p with binding energy values detected at 709.42 eV and 722.14 eV, which correspond to Fe(II)-N compounds [48]. It is noteworthy that ICP1,3 disclose conclusive XPS binding energy values, which confirm their formation (Figures S17 and S18 in the Supporting Information File). Similarly, XPS spectra of OICP1-3 show two main peaks with binding energies for oxygen at ~532 eV, which correspond to oxygen bonded to boron and nitrogen, while the second peak detected at ~533 eV can be assigned to oxygen bonded to sulfur [49]. On top of that, the S2p binding energy of ICP1-3 encountered at ~163 eV was moved to 167 eV in the XPS spectra of OICP1-3 [50], which clearly corroborates the oxidation of the thioether units into their respective sulfone groups (Figures S19–S21 in the Supporting Information File).

### Iodine Adsorption

**Figure 5:**
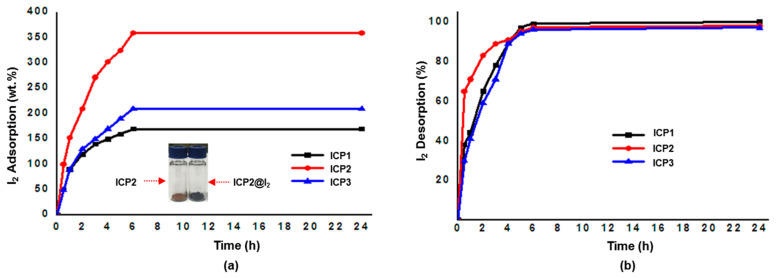
Weight percentage of I_2_ adsorption (**a**) and desorption (**b**) plots of ICP1-3. (Inset: pictures showing the color change before and after I_2_ adsorption).

Iodine uptake capacities of copolymers ICP1-3 and OICP1-3 were investigated employing a typical gravimetric analysis, and the adsorption tests were carried out by placing 10 mg each of copolymers ICP1-3 and OICP1-3 in a glass ampoule, which was kept inside a sealed glass vial which contained surplus solid iodine and heated at 80 °C under atmospheric pressure. Gravimetric analysis allowed for the determination of the mass of iodine adsorbed by each copolymer at different time interludes until attaining equilibrium (Figure 5 and Figure S22 in the Supporting Information File). Table 1 summarizes the wt.% of iodine adsorbed by the target copolymers, which ranges from 170 to 360 wt.%, where the maximum adsorption value was recorded when testing ICP2, i.e., the thioether containing iron(II) clathrochelate copolymer, which bears the bowl-shaped Tröger’s base comonomer units that are assumed to improve iodine capture (Table 1 and Figure 5). This relatively high adsorption recorded for ICP2 promotes it as promising given its versatile synthesis and purification, particularly when related to the compounds stated in the literature, which necessitate multiple synthetic and/or complicated purification steps and yet portray lower iodine adsorption values (Table S2 in the Supporting Information) [23,51,52,53,54,55]. Unsurprisingly, the oxidation of target compounds ICP1-3 into their respective sulfone moieties OICP1-3 either resulted in similar iodine uptakes, namely for OICP1,3 (Table 1 entries 1 and 3), or led to a decrease in the capacity to adsorb iodine, such as the case of OICP2 with an uptake of ~310 wt.% (Table 1 and Figure S22 in the Supporting Information File). It is worthwhile to note that this drop in iodine uptake upon oxidation of thioether into their sulfones was also reported for other polymers [23,51,52,53,54,55,56].

The wt.% of iodine adsorbed by the target copolymers was calculated using the following equation:

(M_2_ − M_1_)/M_1_ × 100% (100 wt% = 1000 mg g^−^^1^) (1)

with M_2_ and M_1_ representing the masses of the copolymer after and before iodine uptake, respectively [57].

**Figure 6:**
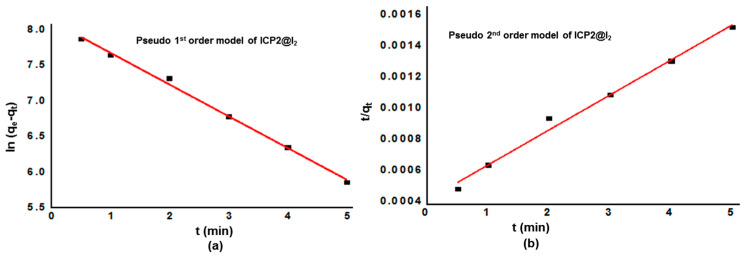
Plots of pseudo-first-order (**a**) and pseudo-second-order (**b**) models of ICP2@I_2_.

The adsorption model of iodine by copolymers ICP1-3 and OICP1-3 was investigated by carrying out kinetic experiments using pseudo-first-order and pseudo-second-order kinetic models (Figure 6 and Figure S23–S27 in the Supporting Information File).

The pseudo-first-order model is expressed by the following equation:

ln(q_e_ − q_t_) = ln q_e_ − k_1_t (2)

Alternatively, the linear equation below was utilized to analyze the pseudo-second-order model:

t/q_t_ = t/q_e_ + 1/k_2_q_e_^2^ (3)

Here, q_t_ (mg g^−^^1^) and q_e_ (mg g^−^^1^) denote the mass of iodine adsorbed per gram adsorbent at time t and at equilibrium, respectively. k_1_ and k_2_ represent the rate constants of the pseudo-first-order and pseudo-second-order models, respectively [58].

As can be perceived from Figure 6, the calculated uptake capacity at equilibrium, q_e_,_cal,_ using the pseudo-first-order model, was obtained by plotting ln(q_e_ − q_t_) versus t. On the other hand, the plot of t/q_t_ versus t was employed to compute q_e,cal_ from the pseudo-second-order model. Table 2 reveals a higher correlation coefficient, R^2^ = 0.9955, which is derived from the linear plot of the pseudo-first-order model, than that derived from the pseudo-second-order model (R^2^ = 0.9880). Moreover, Table 2 discloses the values of the experimental and computed capacities at equilibrium, q_e,exp_ and q_e,cal_, respectively, divulging an improved agreement between the experimental value of 3600 mg g^−^^1^ with the computed capacity at equilibrium extrapolated from the pseudo-first-order model of 3344 mg g^−^^1^. This strongly confirms that iodine uptake by ICP2 follows the pseudo-first-order kinetic model, which would explain its high iodine uptake as opposed to copolymers ICP1,3 and their sulfone-containing derivatives OICP1-3, which were found to follow a pseudo-second-order kinetic model (Figures S23–S27 in the Supporting Information).

**Figure 7:**
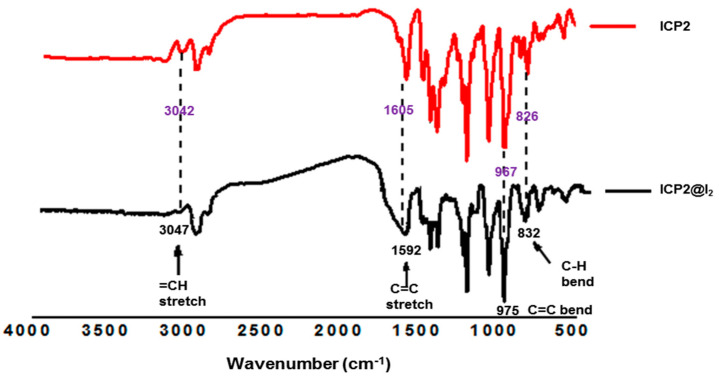
Comparative FTIR spectrum of ICP2 (up) and ICP2@I_2_ (down).

Analysis of iodine adsorption by Fourier transform IR spectroscopy portrays various changes in the vibration bands of the target compounds before and after being loaded with iodine. Figure 7 reveals the comparative Fourier transform IR spectrum of ICP2 against that recorded after it adsorbed iodine, ICP2@I_2_, which clearly discloses the band shifts in the aromatic C=C stretching, alkene C=C bending and aromatic C-H bending vibrations as a result of iodine adsorption. These variations in the FTIR spectrum of ICP2@I_2_ ascertain the interaction between the π-bonds in ICP2 with iodine species [59,60]. The minor change in the peaks also ascertains weak interactions between the copolymer and I_2_, thus suggesting a physisorption of the last onto the surface of ICP2 [61].

**Figure 8:**
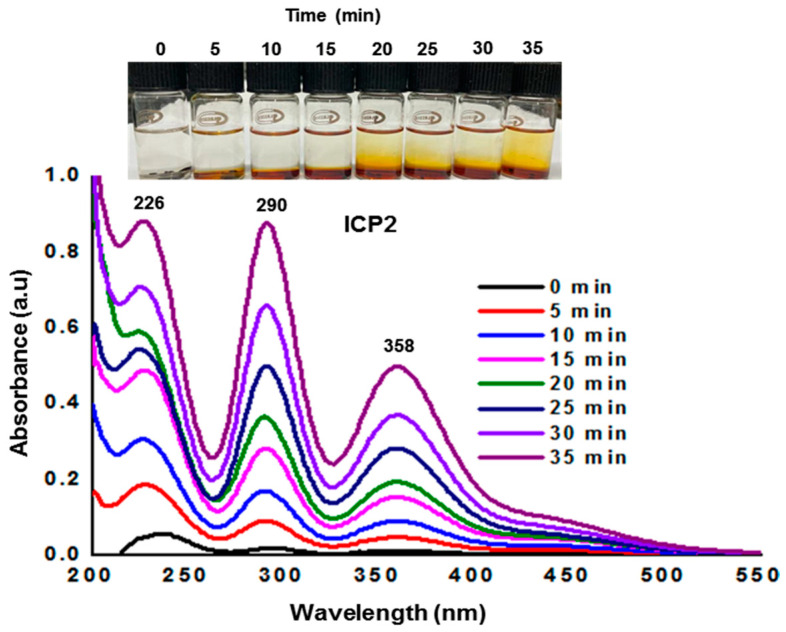
UV-Vis absorption spectra after soaking ICP2@I_2_ in EtOH. (Inset: pictures showing the color change of the solutions upon dipping in EtOH with time).

Copolymers ICP1-3@I_2_ and OICP1-3@I_2_ were heated at 120 °C to extrude I_2,_ where ~ 96% of the latter species were desorbed within 6 *h* for all the target polymers, which released the remaining captured I_2_ by heating them overnight. I_2_ release from the copolymers was explored by dipping ICP2@I_2_ in ethanol, which is known to dissolve iodine, where gradual variation in the color of the medium, from colorless to yellow was noticed, thus implying the release of I_2_ from the copolymer backbone (Figure 8). I_2_ diffusion from ICP2@I_2_ into EtOH was analyzed by UV–Vis spectrophotometry at various time lapses (Figure 8), thus disclosing a noticeable surge in the absorbance maxima corresponding to I_2_, particularly at ~226 nm (typical of I_2_) along with two absorption peaks at ~290 nm and ~358 nm (specific to polyiodide ions), therefore confirming the extrusion of I_2_ from copolymer ICP2 under ambient conditions [62]. After 35 min of soaking the ICP2@I_2_ sample in ethanol, the absorbance intensity did not change, which implied that it reached equilibrium. These observations confirm that the target copolymers can be effortlessly regenerated either by simply heating them in open air or immersing them in ethanol.

**Figure 9:**
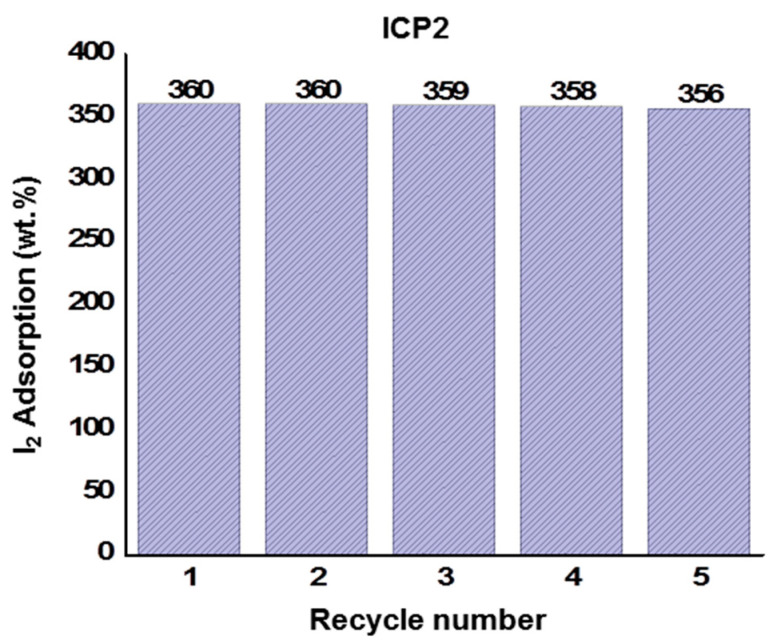
Regeneration performance of iodine adsorption by ICP2.

Reusability tests were carried out using ICP2 as a standard because the latter exhibits the highest I_2_ uptake. Therefore, a completely loaded sample of ICP2 with I_2_, ICP2@I_2_, was heated at 120 °C for 1 day to ensure the full extrusion of the adsorbate, followed by subjecting the regenerated sample ICP2R to I_2_ vapors, and the adsorption values were noted gravimetrically following the same procedure mentioned above. Regeneration tests were repeated for five consecutive adsorption-desorption cycles for ICP2R, which disclosed excellent reusability with only a slight decrease in iodine adsorption efficiency of 1–4% throughout the whole experiment (Figure 9).

## 4. Conclusions

In summary, the one-pot synthesis of three iron(II) clathrochelate-containing copolymers bearing lateral cyclohexyl chains and intercalated by various thioether groups ICP1-3 was reported. The latter endured oxidative reactions of their thioether groups into their respective sulfone moieties, yielding copolymers OICP1-3 in very good yields. I_2_ uptake tests of ICP1-3 and OICP1-3 were carried out, revealing their high uptake capacities reaching a maximum of 3600 mg g^−1^ for ICP2 and whose regeneration of ICP2 proved successful for five successive cycles. Both the modular and eco-friendly synthesis of the metalorganic copolymers presented herein, besides their cost-effectiveness and superior stability, promote them as prominent adsorbents of iodine.

## Figures and Tables

**Table 1:** Summary of iodine adsorption by copolymers ICP1-3 and OICP1-3.

Entry	Copolymer	Wt.% I_2_ Adsorption after 24 h	Oxidized Copolymer	Wt.% I_2_ Adsorption after 24 h
1	ICP1	170	OICP1	180
2	ICP2	360	OICP2	310
3	ICP3	210	OICP3	200

**Table 2:** Pseudo-first-order and pseudo-second-order model parameters for ICP2@I_2_.

Copolymer		Pseudo-First-Order Model			Pseudo-Second-Order Model		
	q_e, exp_ (mg g^−1^)	q_e, cal_ (mg g^−1^)	k_1_ (min^−1^)	R^2^	q_e, cal_ (mg g^−1^)	k_2_ (min^−1^)	R^2^
ICP2@I_2_	3600	3344	−0.0185	0.9955	4454	0.00012	0.9880

## Data Availability

The raw/processed data required to reproduce these findings can be shared upon demand.

## Supplementary Materials

The following Supporting Information can be downloaded at: https://www.mdpi.com/article/10.3390/polym14224818/s1, Figures S1–S3: (1H-NMR spectra of TC, MTC and OMTC); Figures S4–S6: (13C-NMR spectra of TC, MTC and OMTC); Figures S7 and S8: (Solid state 13C-NMR spectra of ICP2,3); Figures S9–S11 (EI-HRMS spectra of TC, MTC and OMTC); Figures S12–S16 (FTIR spectra of MTC, OMTC, ICP1,3 and OICP1,3); Figures S17–S21 (High-resolution XPS spectra of ICP2,3 and OICP1-3); Figure S22 (Iodine adsorption desorption graphs of OICP1-3); Figures S23–S27 (Pseudo 1st and 2nd order model of ICP1,3 and OICP1-3); Table S1 (Summary of iodine adsorption and desorption of ICP1-3 and OICP1-3); Table S2 (Comparison of vapor Iodine adsorption capacity (mg g^−1^) of ICP2 with published adsorbents).
