# Supplementary material for: Synthesis and Iodine Adsorption Properties of Organometallic Copolymers with Propeller-Shaped Fe(II) Clathrochelates Bridged by Different Diaryl Thioether and Their Oxidized Sulfone Derivatives
Source: Polymers (Basel). 2022 Nov 9;14(22):4818. doi: 10.3390/polym14224818 (PMC9697507; doi:10.3390/polym14224818)
Supplement: Supplementary file 1 [file polymers-14-04818-s001.zip › polymers-2012102-supplementary.pdf]

Supporting Information

Synthesis and iodine adsorption properties of organometallic copolymers with propeller-shaped Fe(II) clathrochelates bridged by different diaryl thioether and their oxidized sulfone derivatives

Suchetha Shetty,[1,2] Noorullah Baig,[1,2], and Bassam Alameddine*[1,2]

[1]Department of Mathematics and Natural Sciences, Gulf University for Science and Technology, Kuwait.

[2]Functional Materials Group – GUST, Kuwait.

*Correspondence: alameddine.b@gust.edu.kw

Tel.: +965-2530-7111

Contents

Synthesis of TC	(i)
^{1}H-NMR spectra of TC, MTC and OMTC	Figures S1-S3
^{13}C-NMR spectra of TC, MTC and OMTC	Figures S4-S6
Solid state ^{13}C-NMR spectra of ICP2,3	Figures S7,S8
EI-HRMS spectra of TC, MTC and OMTC	Figures S9-S11
FTIR spectra of MTC, OMTC, ICP1,3 and OICP1,3	Figures S12-S16
High-resolution XPS spectra of ICP2,3 and OICP1-3	Figures S17-S21
Summary of iodine adsorption and desorption of ICP1-3 and OICP1-3	Table S1
Iodine adsorption desorption graphs of OICP1-3	Figure S22
Comparison of vapor Iodine adsorption capacity (mg g-1) of ICP2 with published adsorbents	Table S2
Pseudo 1st and 2nd order model of ICP1,3 and OICP1-3	Figures S23-S27

(i). Synthesis of (E)-(4-((4-(tert-butyl)styryl)thio)phenyl)boronic acid (**TC**)

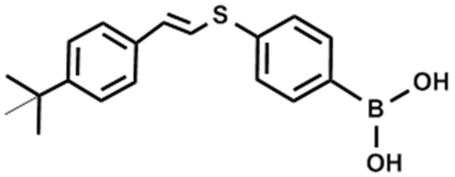

A Schlenk tube was charged with 4-tert-butylphenylacetylene (0.1 g, 0.63 mmol, 1 eq.) and 4-mercaptophenylboronic acid (0.097 g, 0.63 mmol, 1 eq.) in a THF (9 mL). The reaction mixture stirred overnight at 50°C under argon. The resulting solution was precipitated using hexane, followed by filtration affording a white solid (0.187 g, 95%). ^{1}H-NMR (600 MHz, DMSO-d_6, ppm): δ 8.00 (*m*, 2H, -OH), 7.93-7.92 (*brd*, 2H, *J* = 6 *Hz*, ArH), 7.54 (*brm*, 4H, ArH), 7.46 (*m*, 2H, ArH), 6.79-6.78 (*brm*, 2H, Vinylic-CH), 1.39 (*brs*, 9H,t-buyl CH_3); ^{13}C-NMR (150 MHz, DMSO-d_6, ppm): δ 150.31, 135.57, 133.82, 133.27, 128.81, 127.83, 125.66, 123.68, 120.98, 34.83 and 31.50; EI-HRMS: m/z calculated for ($M^{•+}$) $C_{18}H_{21}BO_2S$ 312.1352 found 312.1350

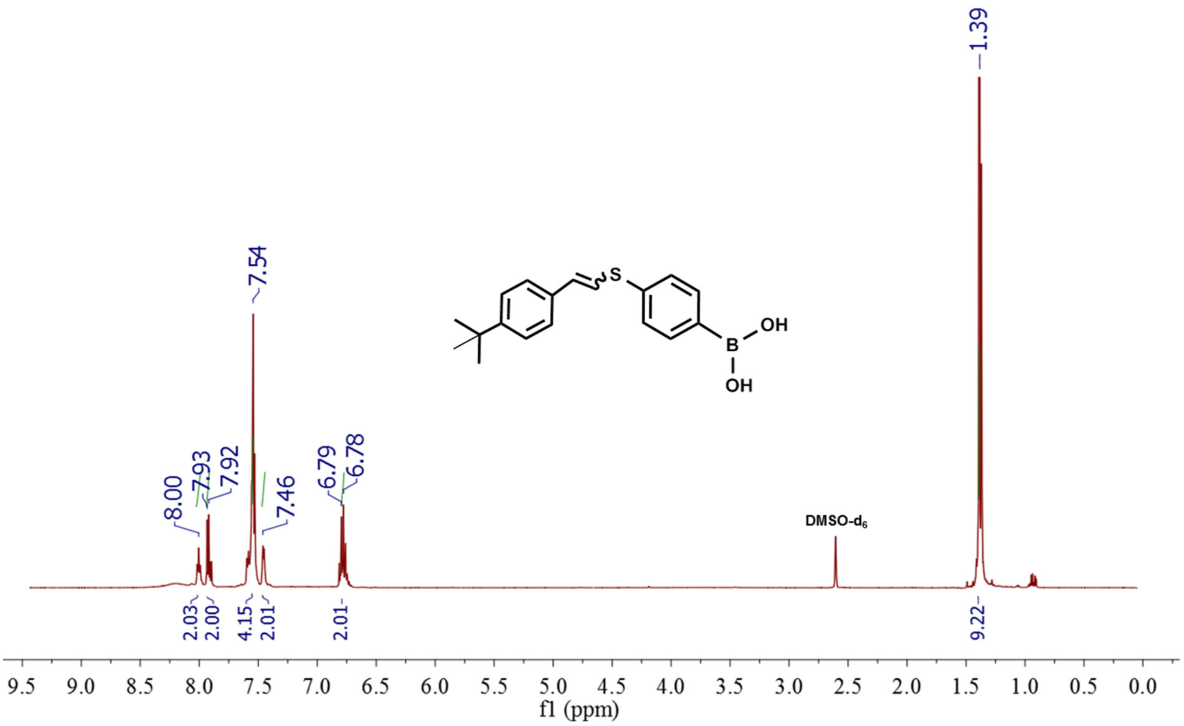

Figure S1 ^{1}H NMR spectrum of **TC**

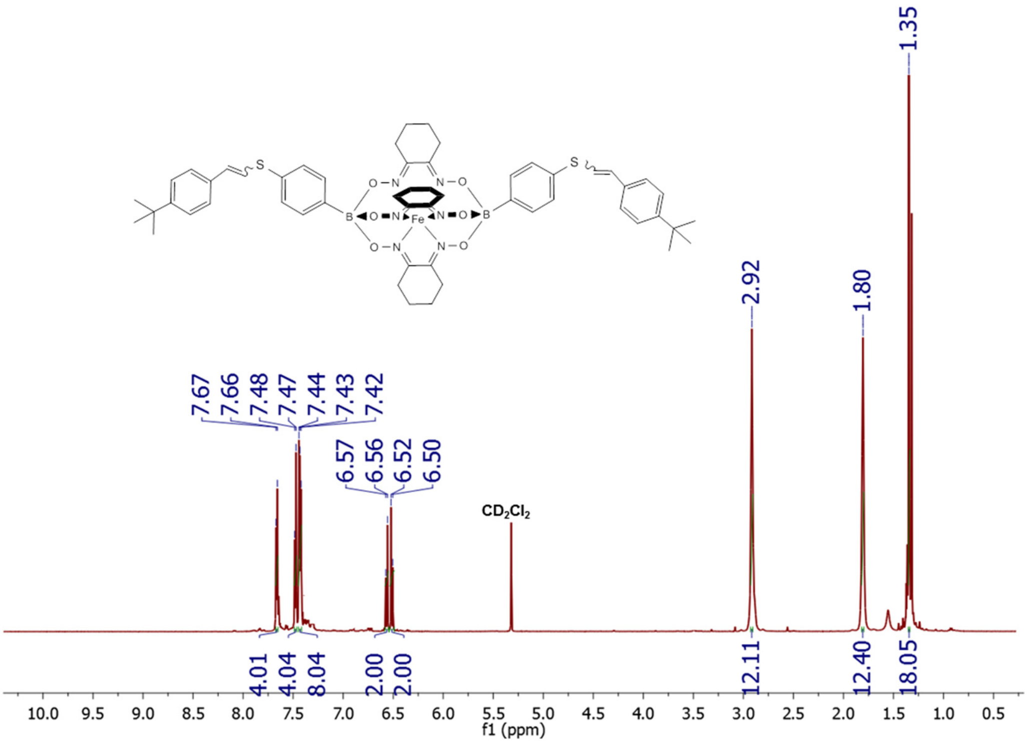

Figure S2 ^{1}H NMR spectrum of **MTC**

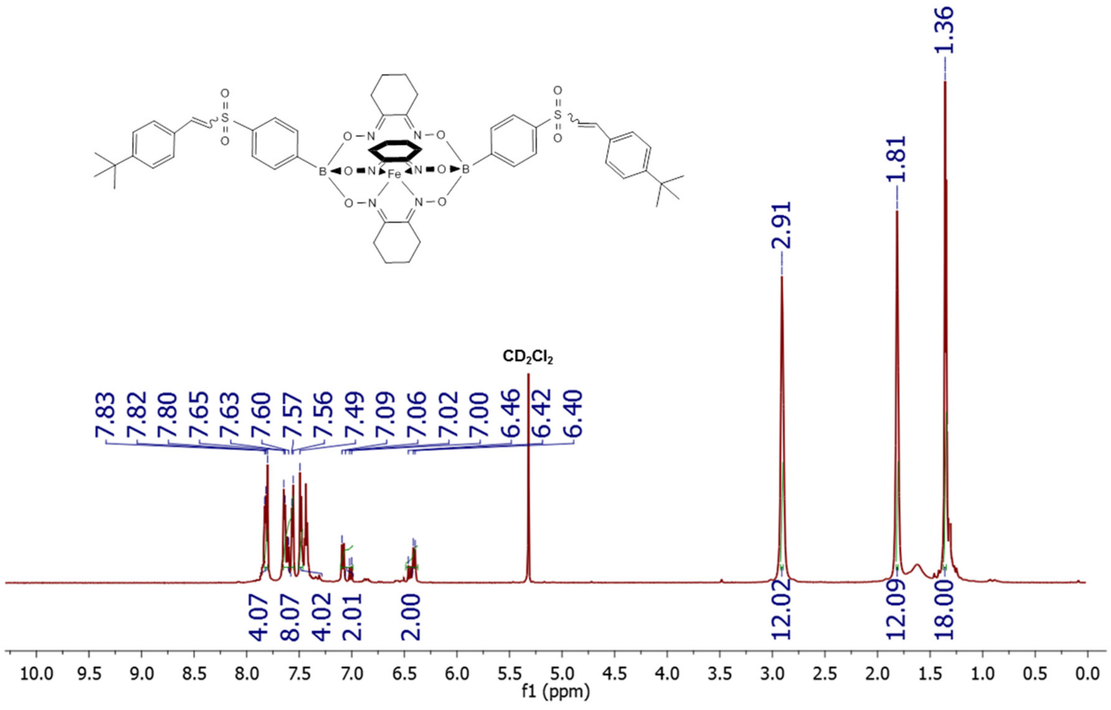

Figure S3 ^{1}H NMR spectrum of **OMTC**

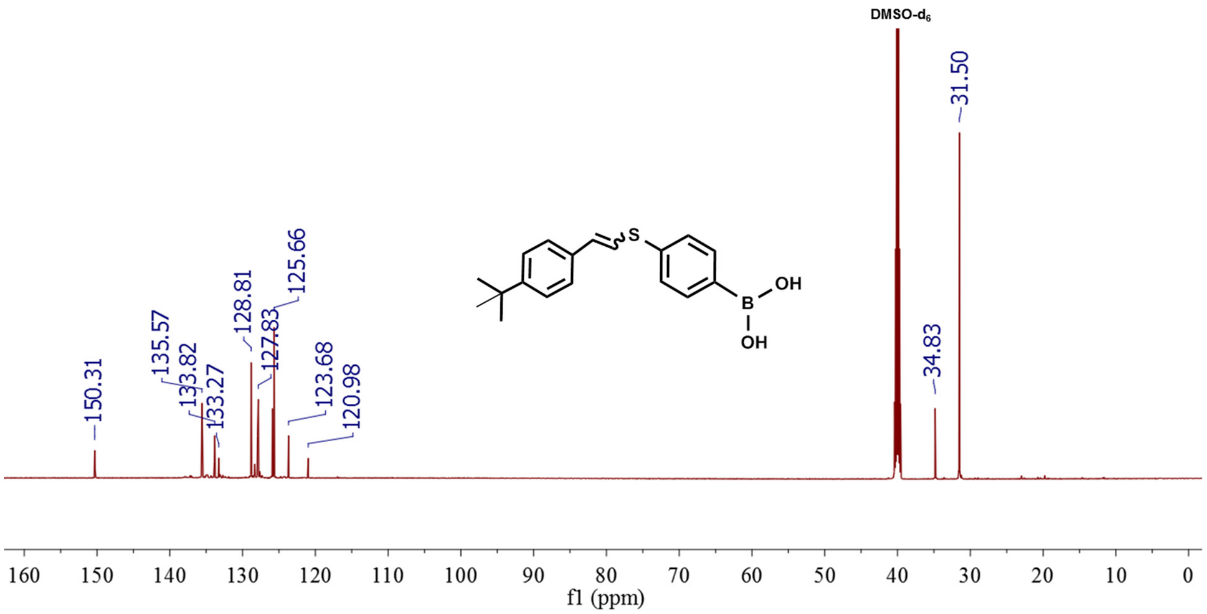

Figure S4 ^{13}C NMR spectrum of **TC**

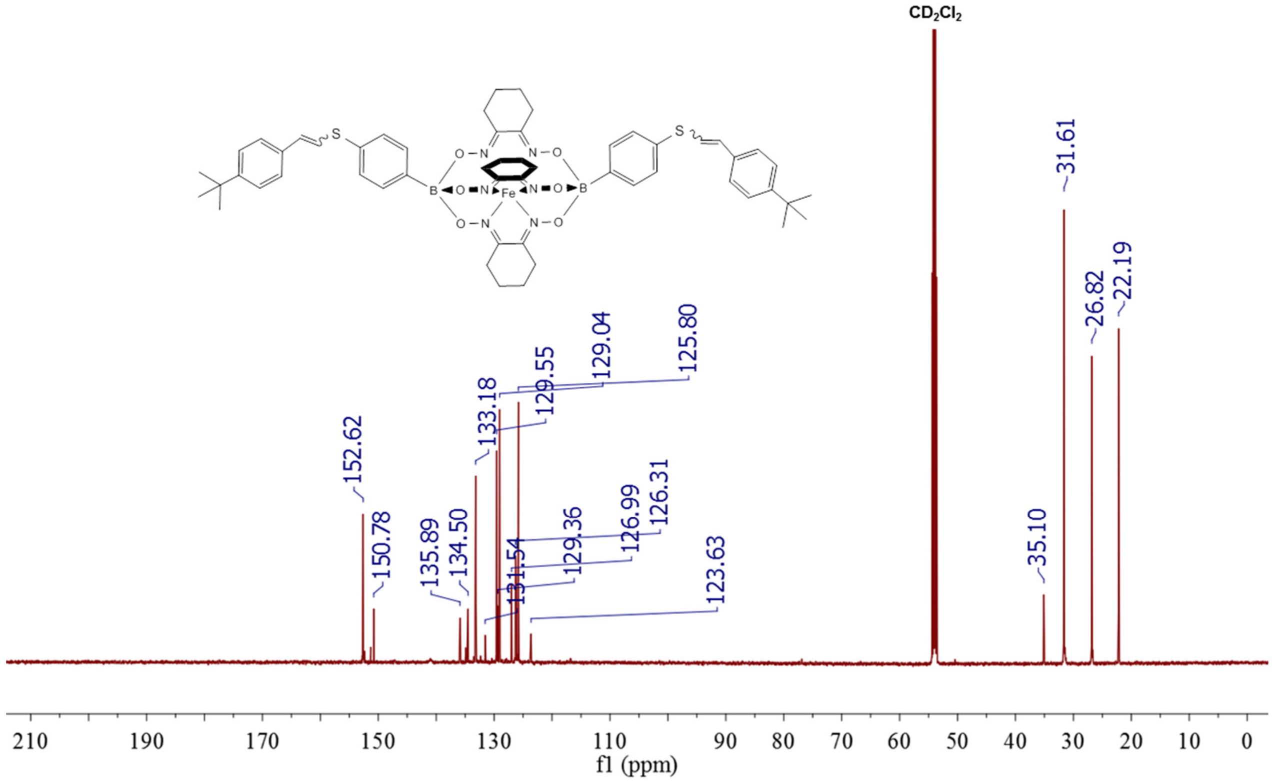

Figure S5 ^{13}C NMR spectrum of **MTC**

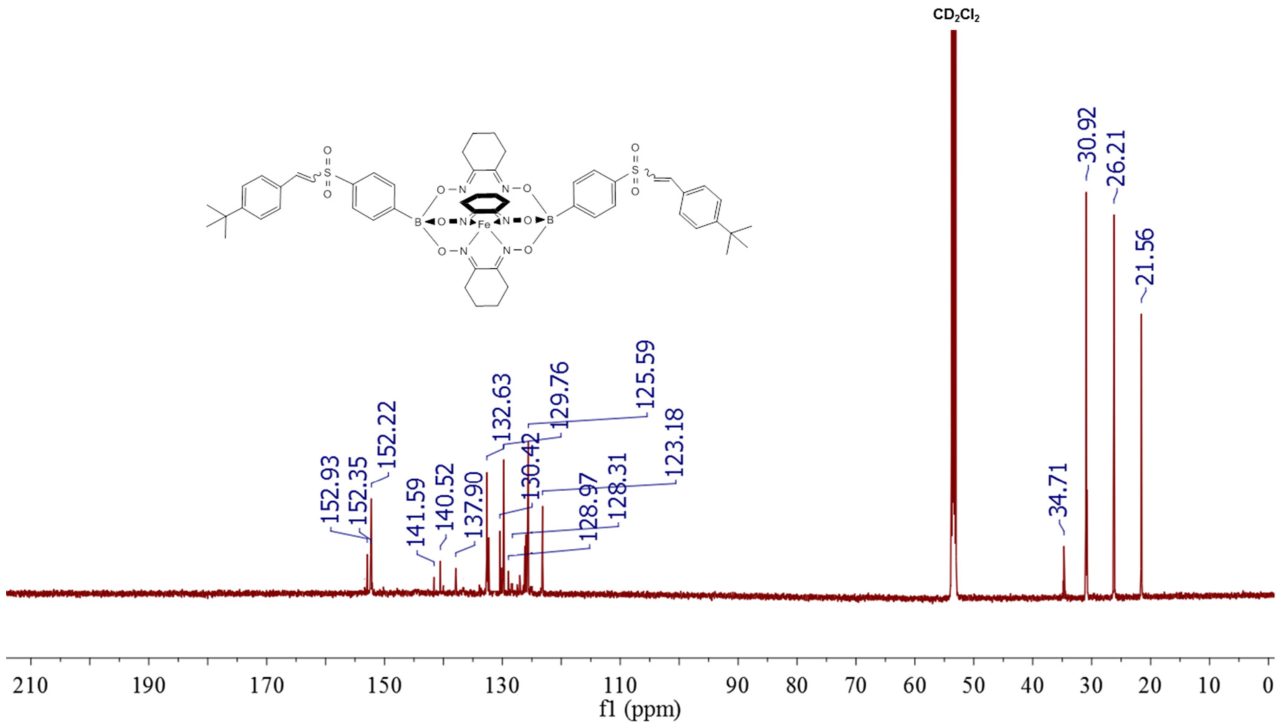

Figure S6 ^{13}C NMR spectrum of **OMTC**

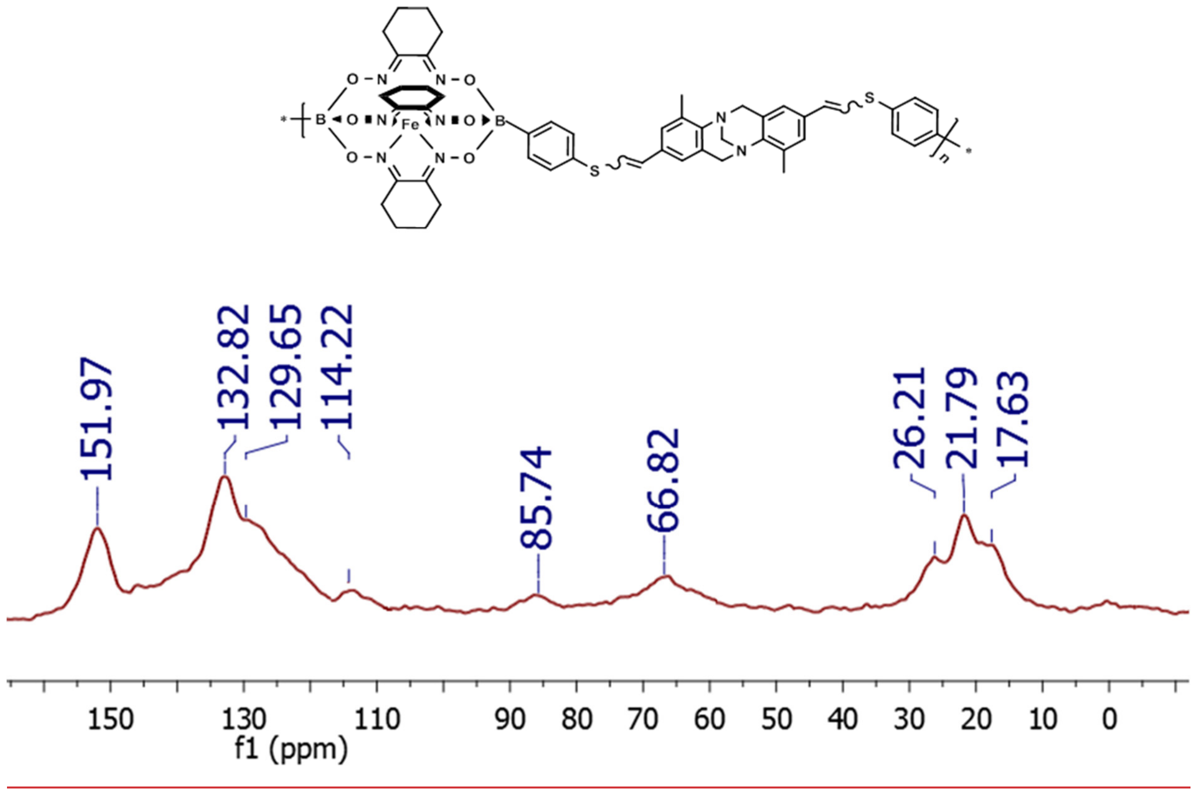

Figure S7 Solid state ^{13}C NMR spectrum of **ICP2**

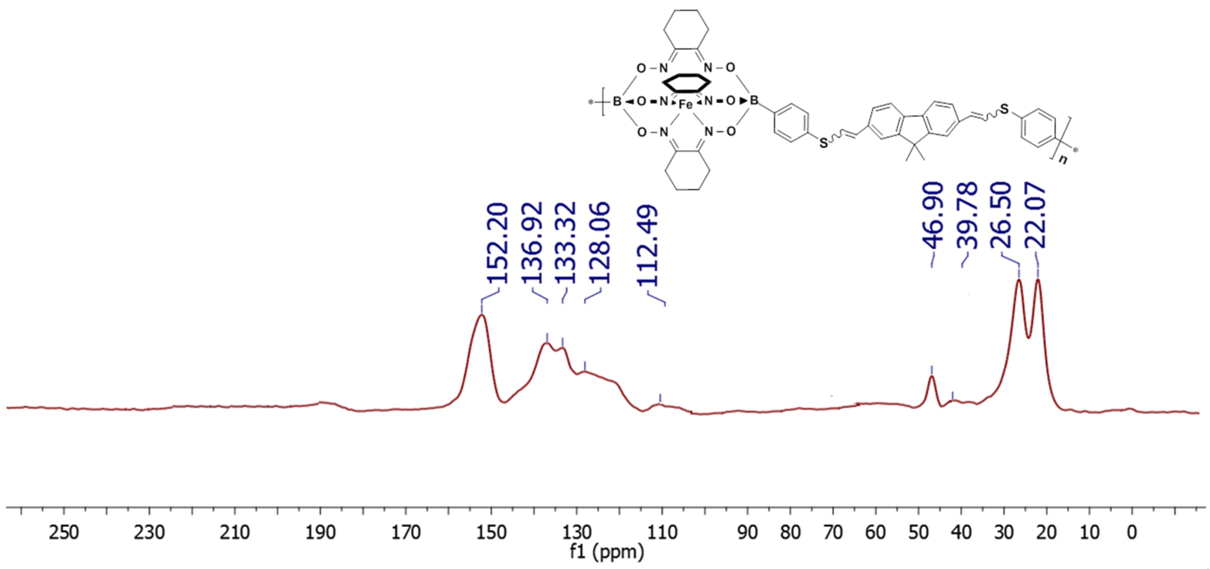

152.20
136.92
133.32
128.06
112.49
46.90
39.78
26.50
22.07
250 230 210 190 170 150 130 110 90 80 70 60 50 40 30 20 10 0
f1 (ppm)

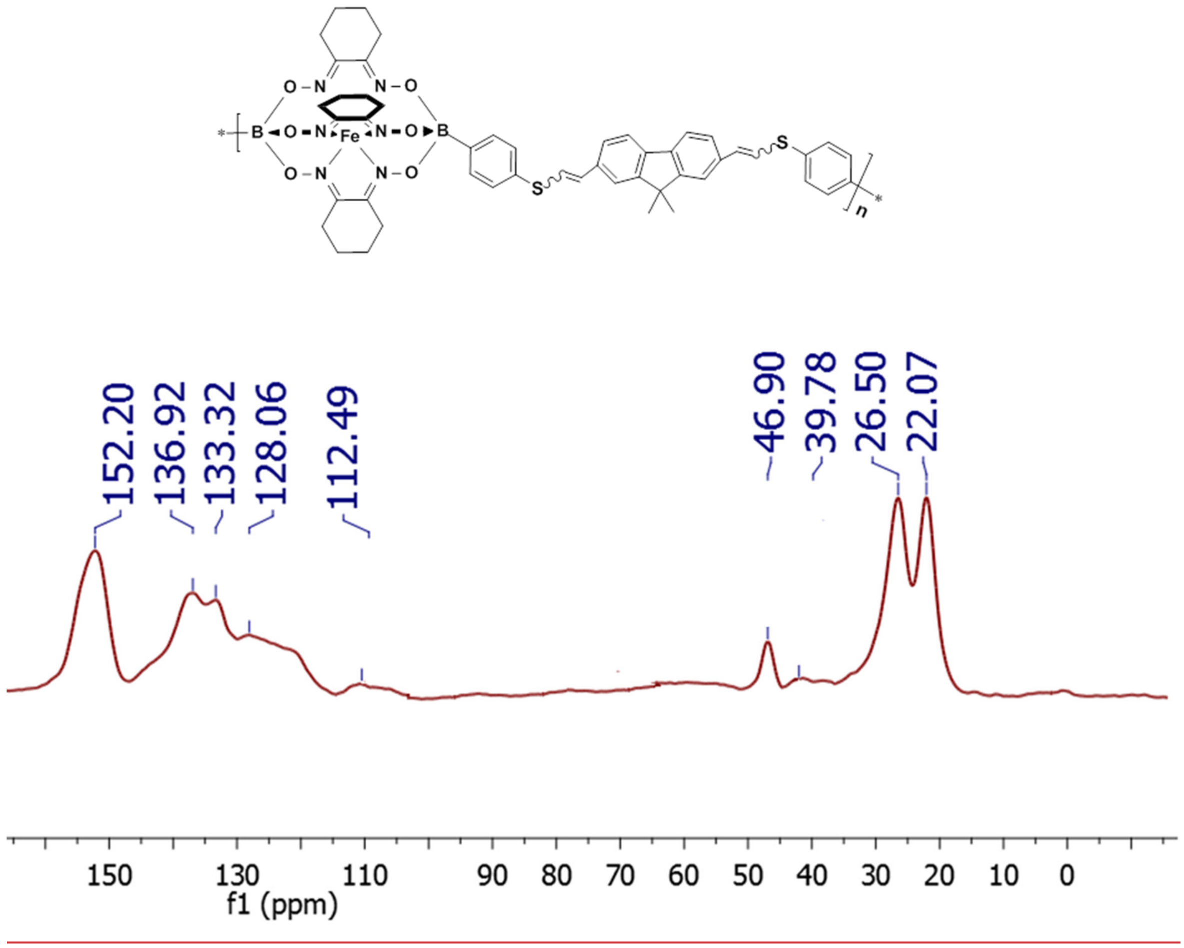

Figure S8 Solid state ^{13}C NMR spectrum of **ICP3**

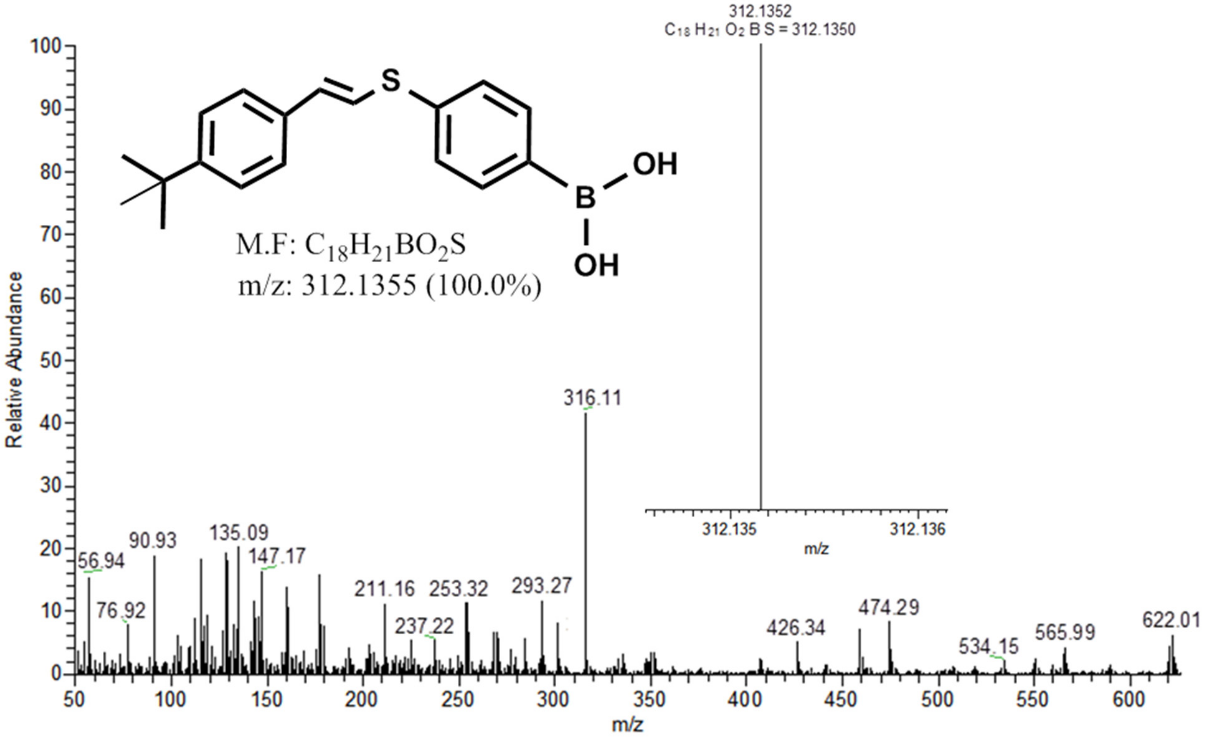

Figure S9 EI-HRMS spectrum of **TC**

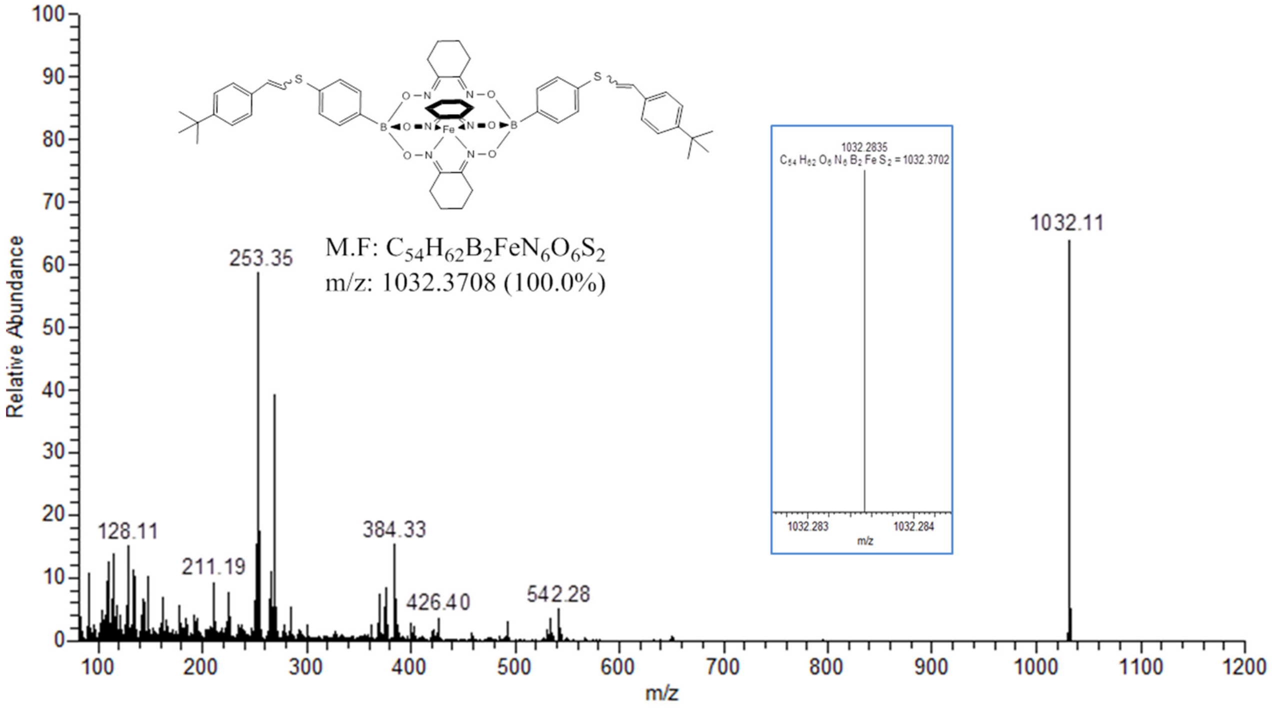

Figure S10 EI-HRMS spectrum of **MTC**

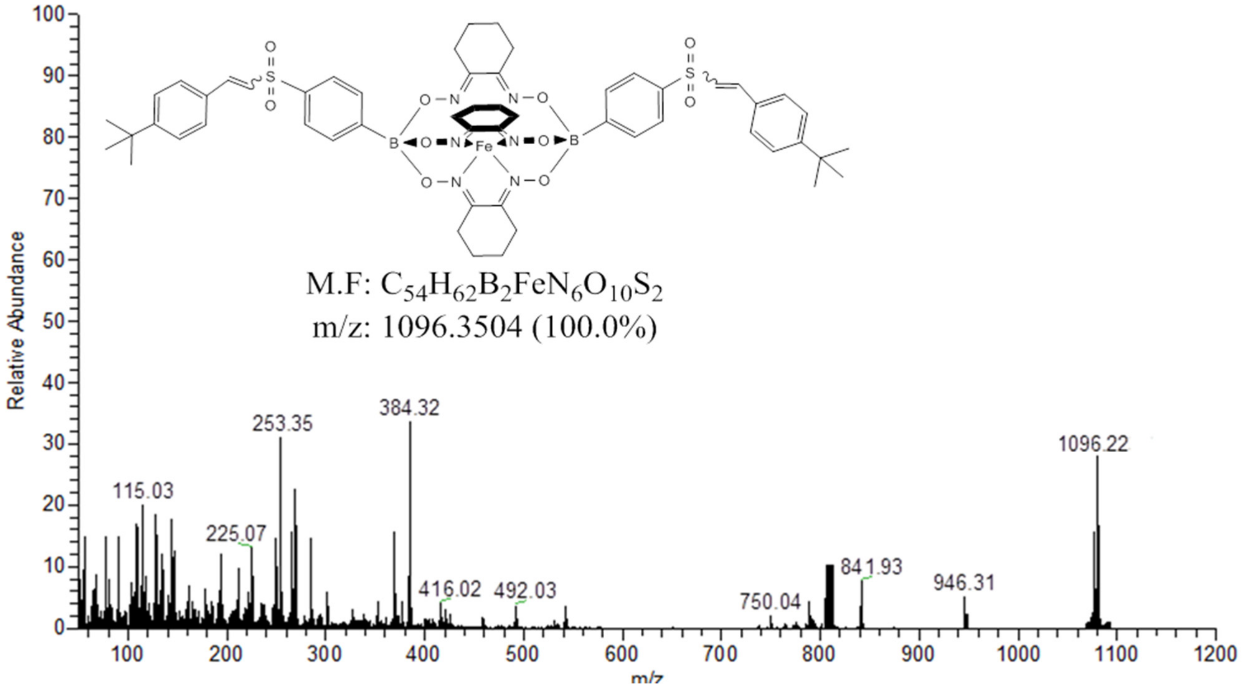

Figure S11 EI-HRMS spectrum of **OMTC**

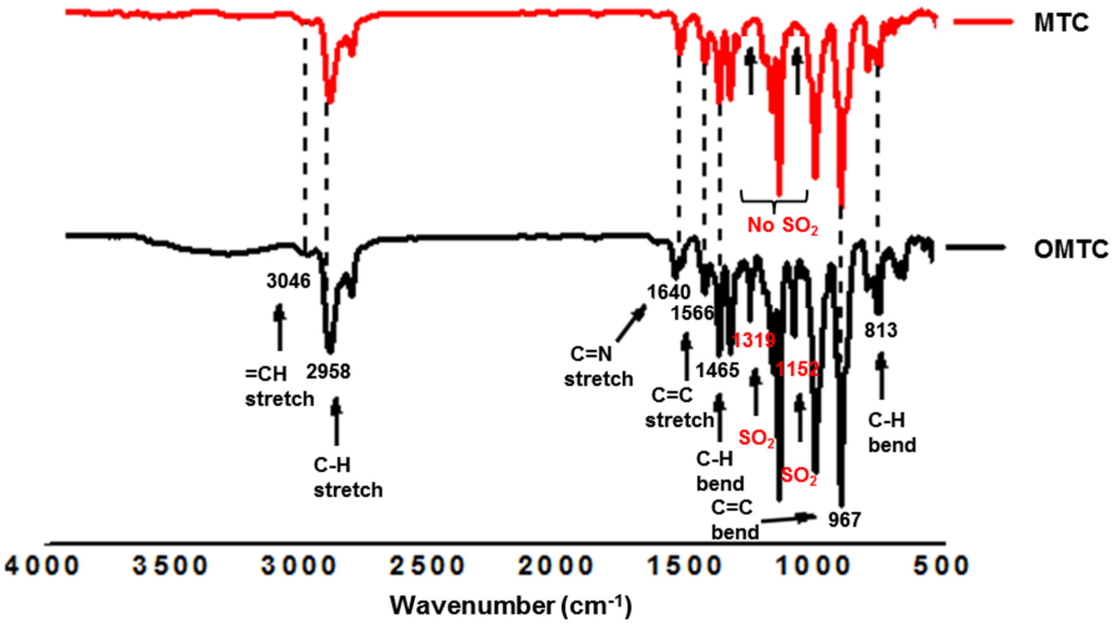

Figure S12 Comparative FTIR spectrum of **MTC** (up) and **OMTC** (down)

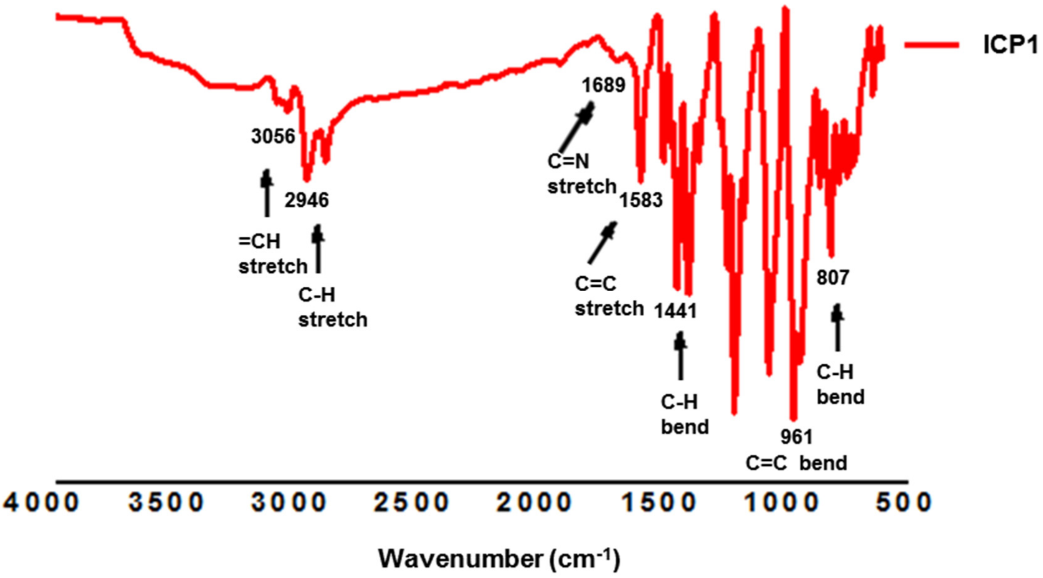

Figure S13 FTIR spectrum of **ICP1**

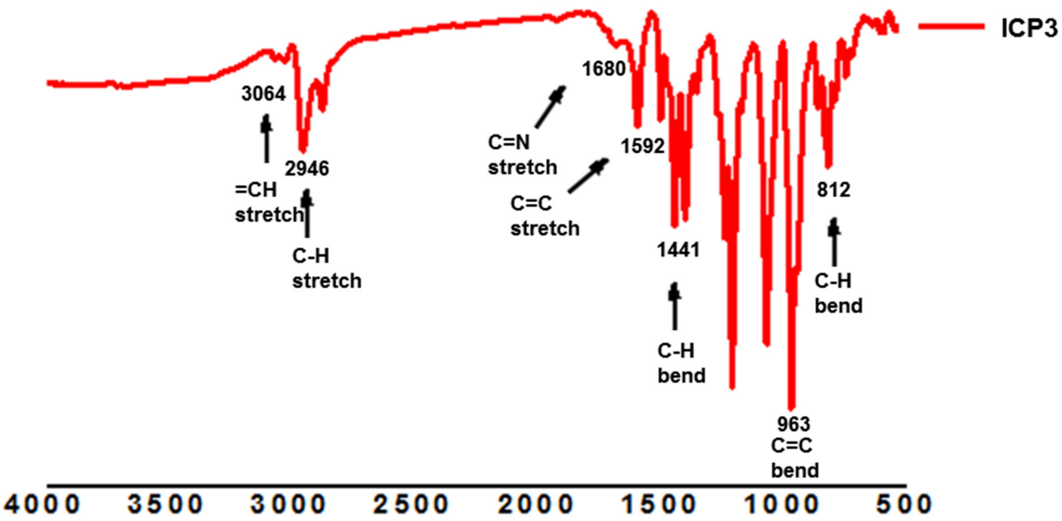

Figure S14 FTIR spectrum of **ICP3**

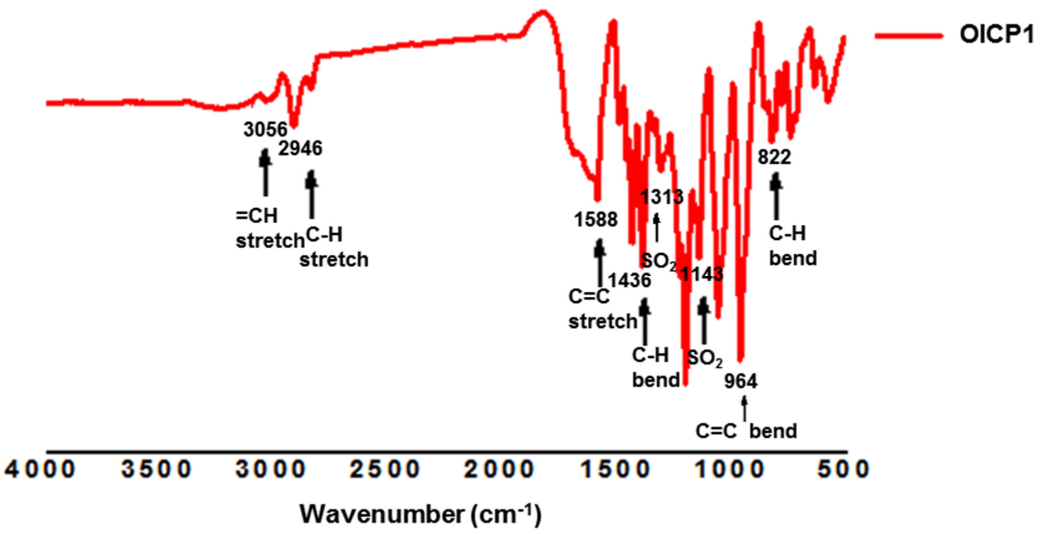

Figure S15 FTIR spectrum of **OICP1**

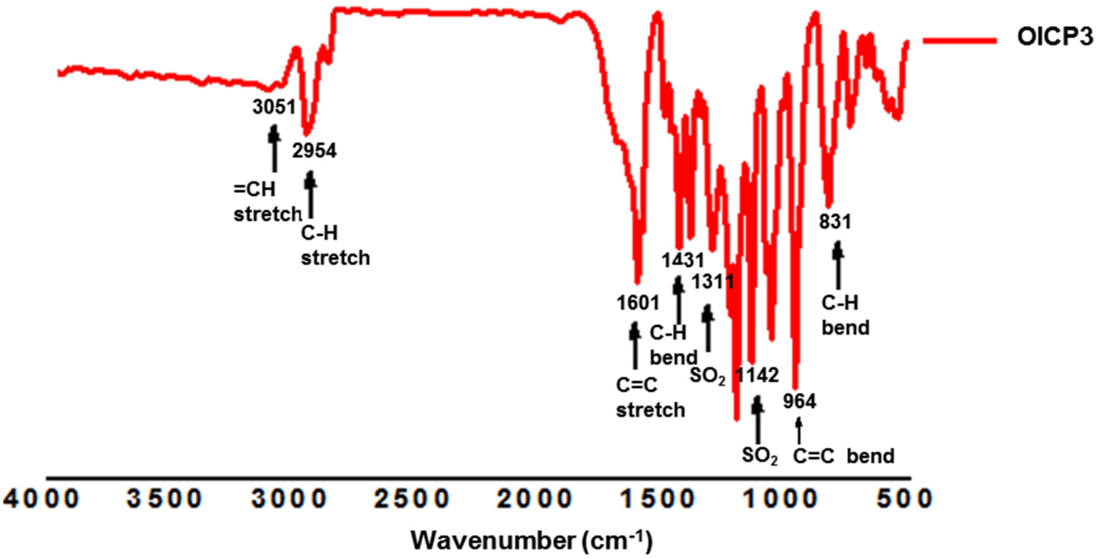

Figure S16 FTIR spectrum of **OICP3**

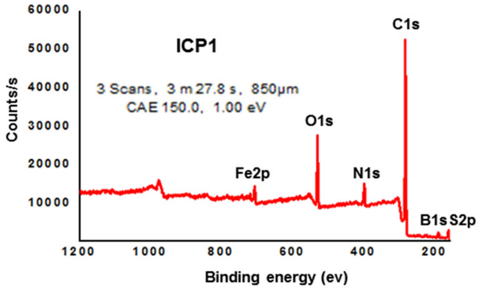

Name	Peak BE
C1s	284.67
C1s A	285.29
O1s	532.47
Fe2p, 3/2	709.40
Fe2p, 1/2	721.96
N1s	400.73
S2p	163.37
B1s	191.04

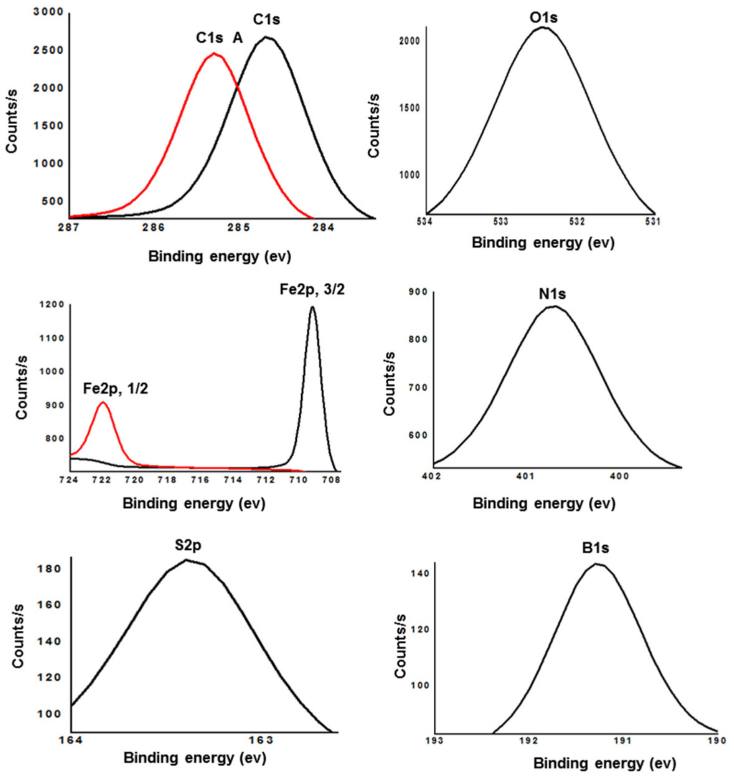

Figure S17 Survey scan-high-resolution XPS spectra of C1s, O1s, Fe2p, N1s, S2p and B1s of **ICP1**

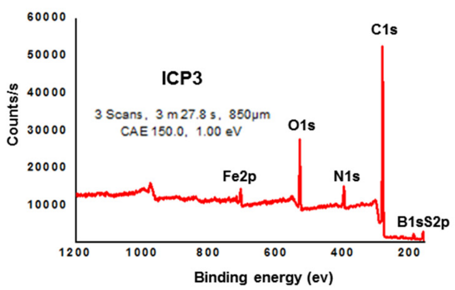

Name	Peak BE
C1s	284.66
C1s A	285.39
O1s	532.70
Fe2p, 3/2	709.45
Fe2p, 1/2	722.11
N1s	400.74
S2p	163.62
B1s	191.32

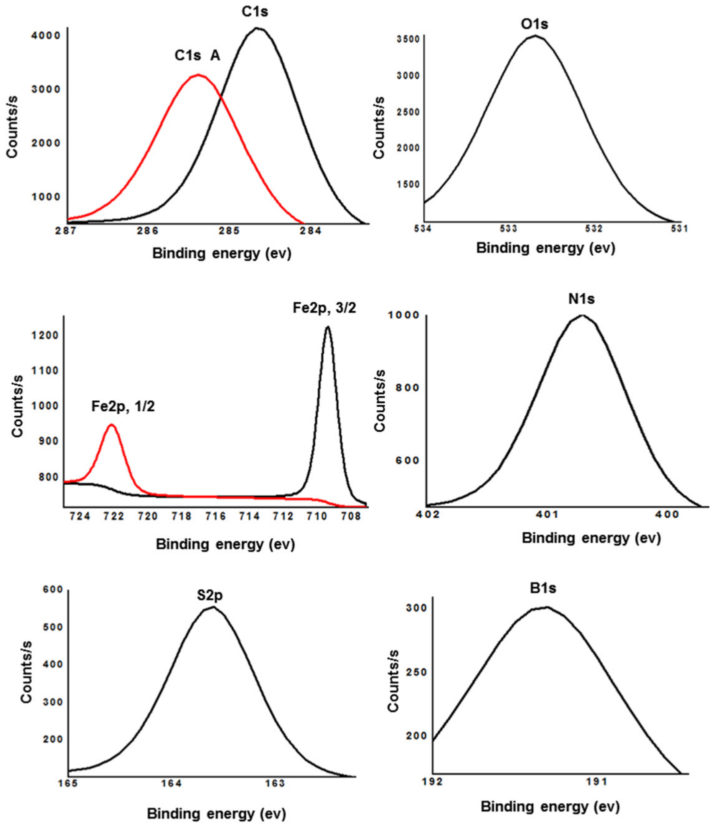

Figure S18 Survey scan-high-resolution XPS spectra of C1s, O1s, Fe2p, N1s, S2p and B1s of **ICP3**

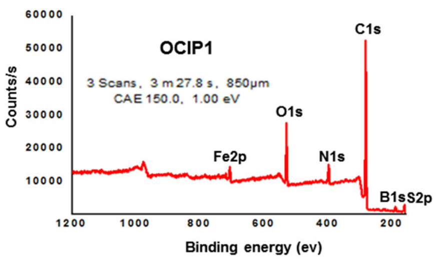

Name	Peak BE
C1s	284.63
C1s A	285.36
O1s	532.47
O1S A	533.36
N1s	400.63
S2p	167.90
Fe2p, 3/2	709.29
Fe2p, 1/2	722.00
B1s	191.32

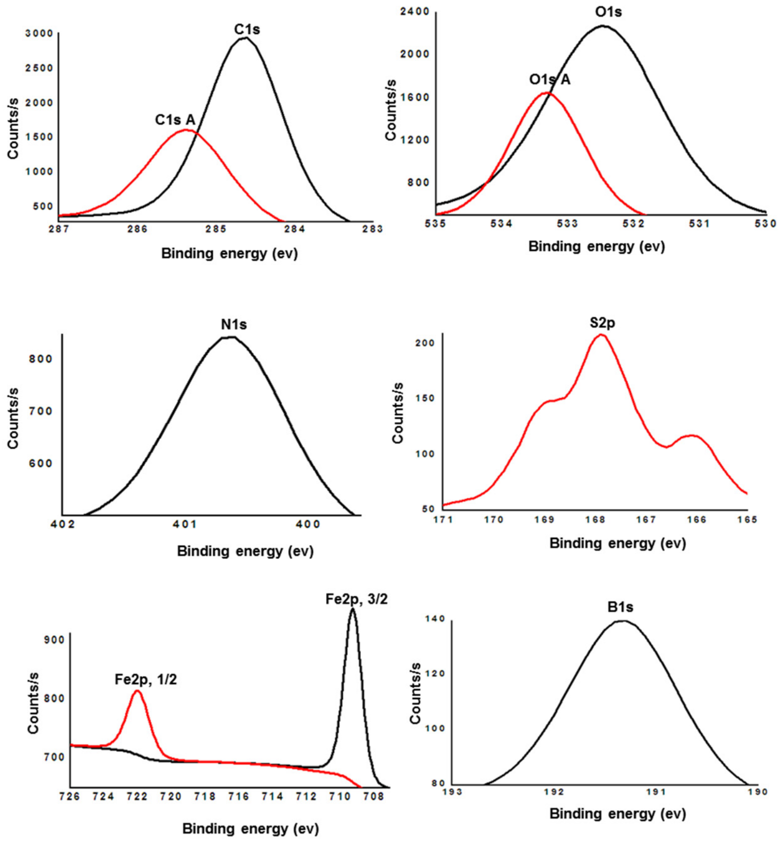

Figure S19 Survey scan-high-resolution XPS spectra of C1s, O1s, N1s, S2p, Fe2p and B1s of **OICP1**

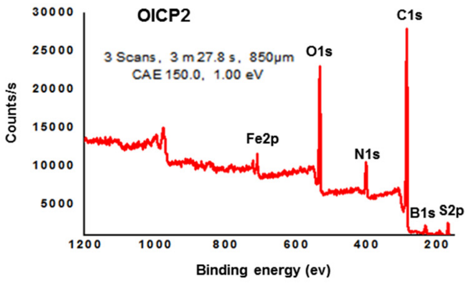

Name	Peak BE
C1s	284.61
C1S A	285.83
O1s	532.06
O1S A	532.93
N1s	400.09
N1s A	398.62
S2p	167.37
Fe2p, 3/2	708.90
Fe2p, 1/2	721.53
B1s	190.73

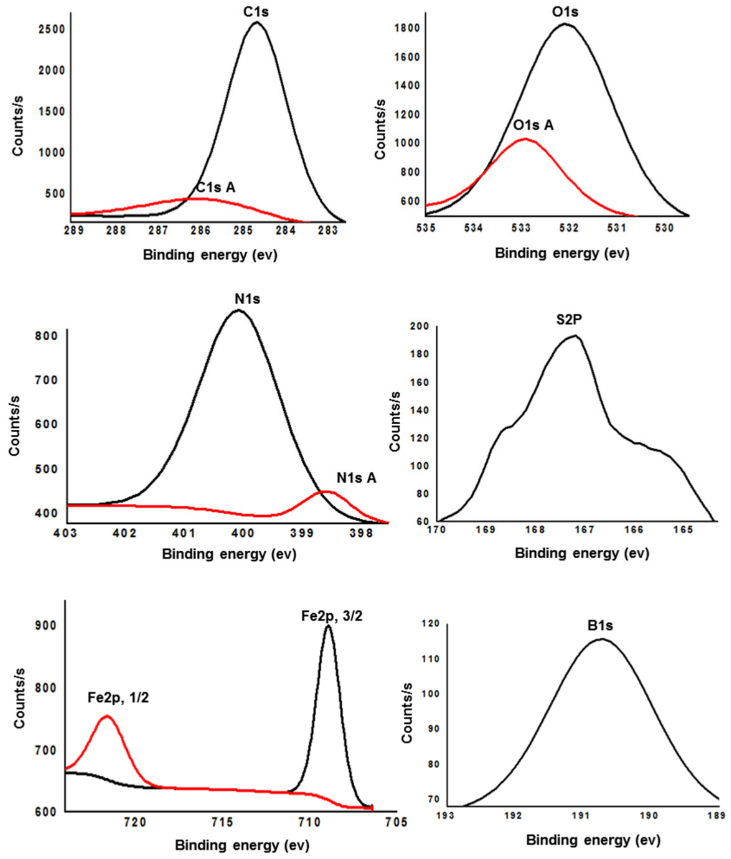

Figure S20 Survey scan-high-resolution XPS spectra of C1s, O1s, N1s, S2p, Fe2p and B1s of **OICP2**

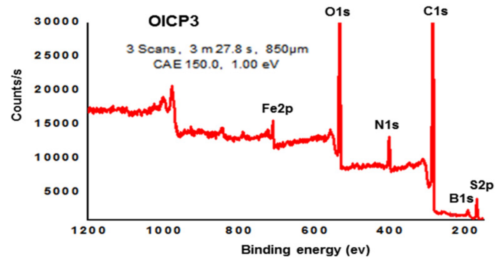

Name	Peak BE
C1s	284.64
C1s A	285.26
O1s	532.48
O1s A	533.54
N1s	400.82
S2p	167.94
Fe2p, 3/2	709.54
Fe2p, 1/2	722.24
B1s	191.41

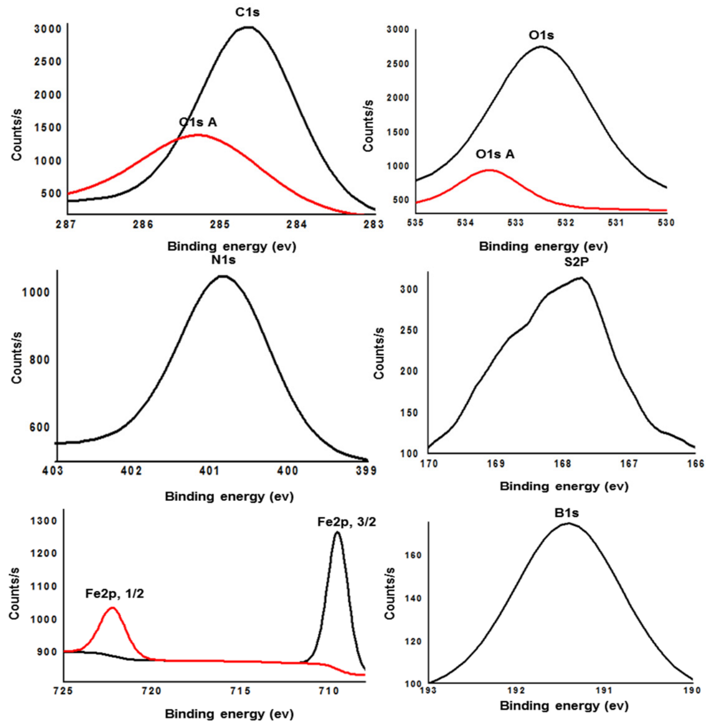

Figure S21 Survey scan-high-resolution XPS spectra of C1s, O1s, N1s, S2p, Fe2p and B1s of **OICP3**

Table S1 Summary of iodine adsorption and desorption of copolymers ICP1-3 and OICP1-3

Entry	Polymer	Wt.% I_2 adsorption after 24 h	% I_2 desorption after 24 h
1	**ICP1**	170	100
2	**ICP2**	360	98
3	**ICP3**	210	97
4	**OICP3**	180	98
5	**OICP2**	310	99
6	**OICP3**	200	97

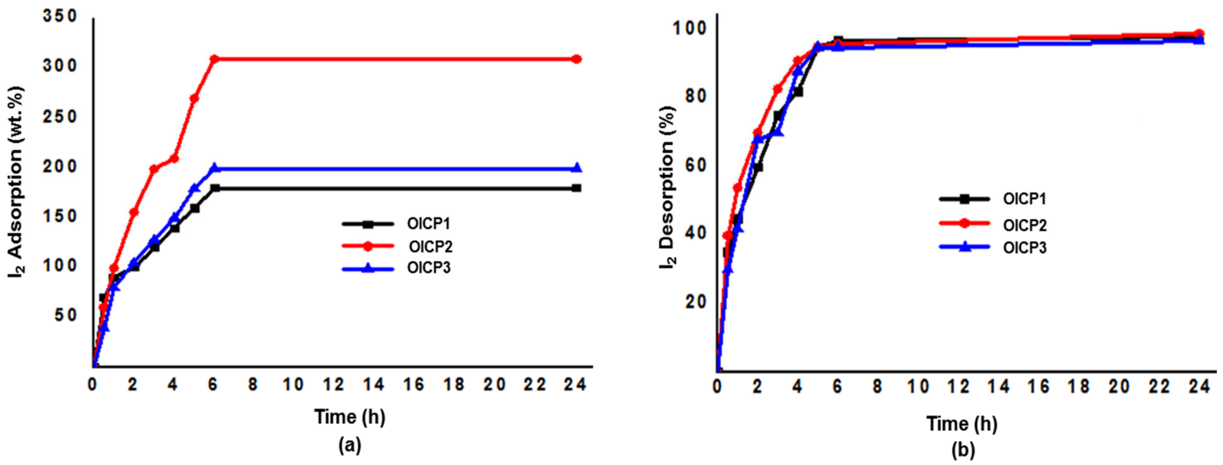

Figure S22 Wt% of iodine adsorption (**a**) and % of iodine desorption (**b**) graphs of **OICP1-3.**

Table S2 Comparison of vapor iodine adsorption capacity (mg g^{-1}) of ICP2 with other reported adsorbents

Entry	Adsorbents	Adsorption Capacity (mg g^{-1})	Reference
1	MOF nanosheets	3510	Yu, Inorg. Chem. 2022
2	NH-COF	2600	Mokhtari, Sep. Purif. Technol. 2022
3	Thorium–organic nanotube	955	Hastings, Inorg. Chem.2022
4	Bitumite-based HPC	924	Yin, RSC Advs.2022
5	Polyhedral silsesquioxane materials	363	Gamal, Polymers 2021
6	Cu_2O/TMU-17-NH_2 composite	300	Yadollahi, J. Hazard. Mater.2020
7	ICP2	3600	Present work

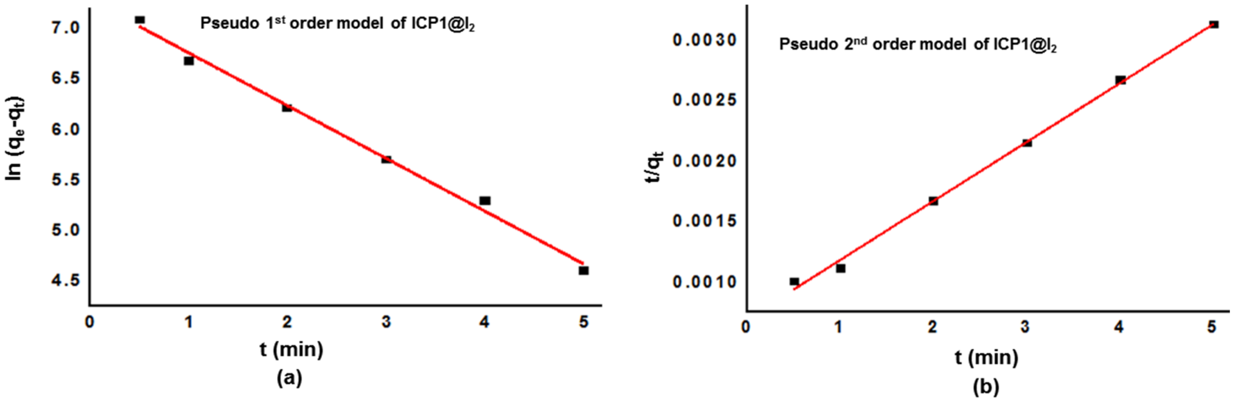

Copolymer	Pseudo 1st order model				Pseudo 2nd order model		
	$q_{e, exp}$ (mg g^{-1})	$q_{e, cal}$ (mg g^{-1})	k_1 (min^{-1})	R^2	$q_{e, cal}$ (mg g^{-1})	k_2 (min^{-1})	R^2
**ICP1@I$_2$**	1700	1460	-0.02183	0.9921	2083	0.000332	0.9967

Figure S23 Pseudo 1st and 2nd order model of **ICP1@I$_2$**

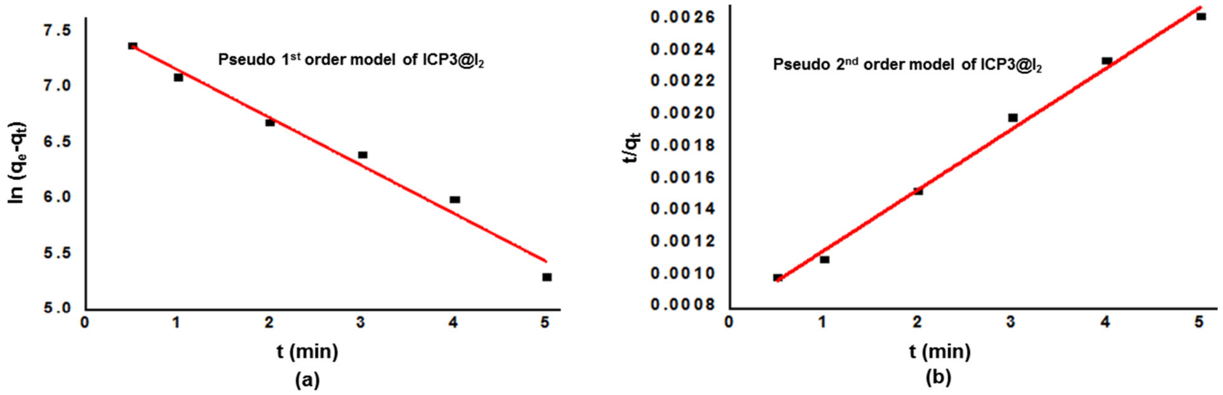

Copolymer		Pseudo 1st order model			Pseudo 2nd order model		
	$q_{e, exp}$ (mg g^{-1})	$q_{e, cal}$ (mg g^{-1})	k_1 (min^{-1})	R^2	$q_{e, cal}$ (mg g^{-1})	k_2 (min^{-1})	R^2
ICP3@I_2	2100	1966	-0.01791	0.9777	2631	0.000183	0.99202

Figure S24 Pseudo 1st and 2nd order model of **ICP3@I_2**

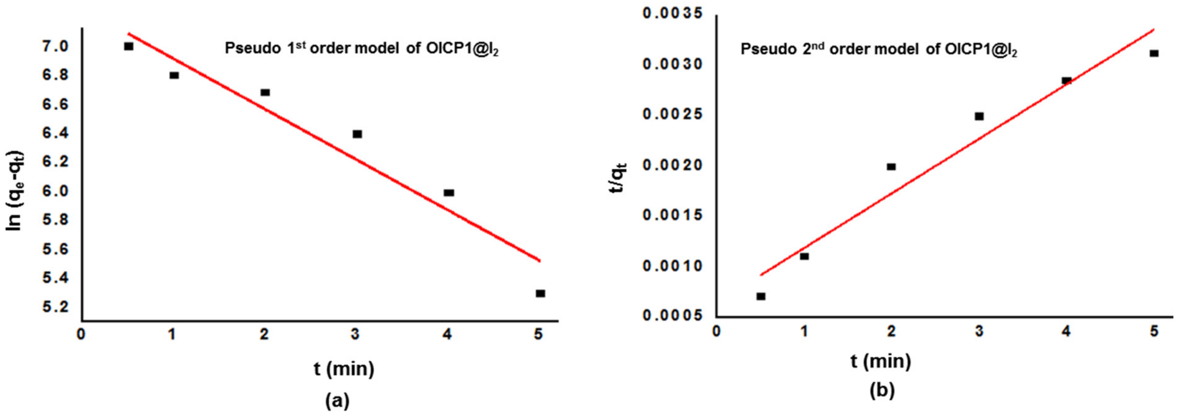

Copolymer	Pseudo 1st order model				Pseudo 2nd order model		
	$q_{e, exp}$ (mg g^{-1})	$q_{e, cal}$ (mg g^{-1})	k_1 (min^{-1})	R^2	$q_{e, cal}$ (mg g^{-1})	k_2 (min^{-1})	R^2
**OICP1@I$_2$**	1800	1427	-0.01453	0.9175	1848	0.00045	0.9394

Figure 25 Pseudo 1st and 2nd order model of **OICP1@I$_2$**

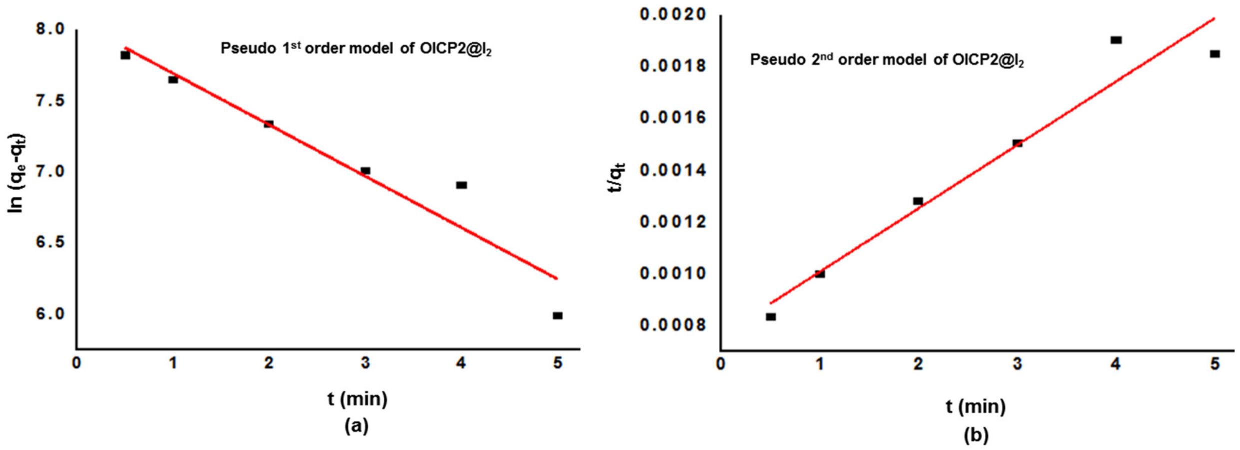

Copolymer	Pseudo 1st order model				Pseudo 2nd order model		
	$q_{e,\,exp}$ (mg g^{-1})	$q_{e,\,cal}$ (mg g^{-1})	k_1 (min^{-1})	R^2	$q_{e,\,cal}$ (mg g^{-1})	k_2 (min^{-1})	R^2
**OICP2@I$_2$**	3100	3153	-0.01509	0.90652	4072	0.000079	0.9471

Figure 26 Pseudo 1st and 2nd order model of **OICP2@I$_2$**

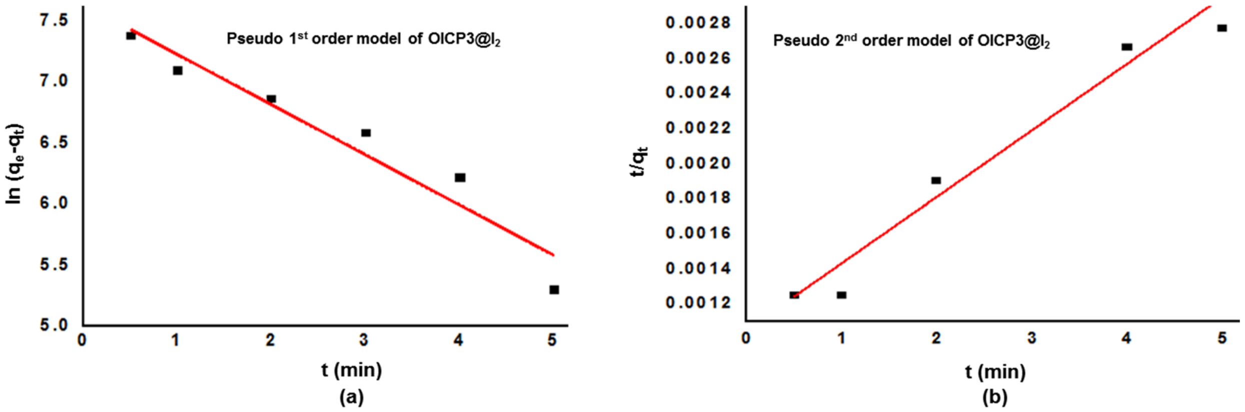

Copolymer	Pseudo 1st order model				Pseudo 2nd order model		
	$q_{e, exp}$ (mg g^{-1})	$q_{e, cal}$ (mg g^{-1})	k_1 (min^{-1})	R^2	$q_{e, cal}$ (mg g^{-1})	k_2 (min^{-1})	R^2
**OICP3@I_2**	2000	2060	-0.01711	0.9167	2660	0.000135	0.9433

Figure 27 Pseudo 1st and 2nd order model of **OICP3@I_2**
